# N^6^-Methyladenosine RNA Modification: An Emerging Immunotherapeutic Approach to Turning Up Cold Tumors

**DOI:** 10.3389/fcell.2021.736298

**Published:** 2021-09-20

**Authors:** Lei Zhan, Junhui Zhang, Suding Zhu, Xiaojing Liu, Jing Zhang, Wenyan Wang, Yijun Fan, Shiying Sun, Bing Wei, Yunxia Cao

**Affiliations:** ^1^Department of Obstetrics and Gynecology, The First Affiliated Hospital of Anhui Medical University, Hefei, China; ^2^Department of Obstetrics and Gynecology, The Second Affiliated Hospital of Anhui Medical University, Hefei, China; ^3^NHC Key Laboratory of Study on Abnormal Gametes and Reproductive Tract, Anhui Medical University, Hefei, China; ^4^Key Laboratory of Population Health Across Life Cycle, Ministry of Education of the People’s Republic of China, Anhui Medical University, Hefei, China

**Keywords:** N^6^-methyladenosine RNA modification, tumor microenvironment, cold tumors, hot tumors, biomarker, prognosis, immunotherapy

## Abstract

Immunotherapy is a novel clinical approach that has shown clinical efficacy in multiple cancers. However, only a fraction of patients respond well to immunotherapy. Immuno-oncological studies have identified the type of tumors that are sensitive to immunotherapy, the so-called hot tumors, while unresponsive tumors, known as “cold tumors,” have the potential to turn into hot ones. Therefore, the mechanisms underlying cold tumor formation must be elucidated, and efforts should be made to turn cold tumors into hot tumors. N^6^-methyladenosine (m^6^A) RNA modification affects the maturation and function of immune cells by controlling mRNA immunogenicity and innate immune components in the tumor microenvironment (TME), suggesting its predominant role in the development of tumors and its potential use as a target to improve cancer immunotherapy. In this review, we first describe the TME, cold and hot tumors, and m^6^A RNA modification. Then, we focus on the role of m^6^A RNA modification in cold tumor formation and regulation. Finally, we discuss the potential clinical implications and immunotherapeutic approaches of m^6^A RNA modification in cancer patients. In conclusion, m^6^A RNA modification is involved in cold tumor formation by regulating immunity, tumor-cell-intrinsic pathways, soluble inhibitory mediators in the TME, increasing metabolic competition, and affecting the tumor mutational burden. Furthermore, m^6^A RNA modification regulators may potentially be used as diagnostic and prognostic biomarkers for different types of cancer. In addition, targeting m^6^A RNA modification may sensitize cancers to immunotherapy, making it a promising immunotherapeutic approach for turning cold tumors into hot ones.

## Introduction

Cancer currently ranks as one of the leading causes of death worldwide, and the latest reports indicate that the number of cancer patients is expected to rise by 70% in the next two decades (World Health Organization, 2014). Tumor development depends on the sophisticated tumor microenvironment (TME), which includes tumor, stromal, and immune cells as well as non-cellular components, such as vascular structure (Duan et al., 2020). Traditional chemoradiotherapy focuses on targeting tumor cells; in contrast, immunotherapy aims to activate immune cells and has emerged as an approach capable of achieving remarkable advances in cancer treatment (Lohmueller and Finn, 2017; Simone, 2020). Currently, immune checkpoint inhibitors targeting cytotoxic T-cell lymphocyte-associated protein 4 (CTLA-4), programmed death receptor 1 (PD-1), and the ligand PD-L1 have been approved by the Food and Drug Administration (FDA) (Rotte, 2019; Aggen et al., 2020; Vaddepally et al., 2020). Furthermore, other kinds of immune checkpoint inhibitors are currently under investigation, such as lymphocyte activation gene-3 (LAG-3), T-cell immunoglobulin and mucin-domain containing-3 (TIM-3), T-cell immunoglobulin and ITIM domain (TIGIT), and V-domain Ig suppressor of T-cell activation (VISTA) (Qin et al., 2019). Nevertheless, a large fraction of patients do not respond to immunotherapy. Importantly, studies exploring the TME have identified the kind of patients that are more sensitive to immunotherapy (Galon and Bruni, 2019). Briefly, depending on the response rates to immunotherapy, tumors are commonly divided into “hot tumors,” whose TME is characterized by the presence of tumor-infiltrating lymphocytes (TILs) and molecular signatures of immune activation, and “cold tumors,” whose TME is characterized by the absence of TILs and neoantigens (Galon et al., 2007; Camus et al., 2009; Van Allen et al., 2015; Gajewski et al., 2017; Huang et al., 2017). Consequently, numerous studies have aimed to turn cold tumors into hot ones (Rosenberg and Restifo, 2015; Sharma and Allison, 2015). For instance, recruitment of CD8^+^ T cells into cold tumors by rescuing interferon γ (IFN-γ) improves the immunopotentiating effect of dendritic cells (DCs) (Li X. et al., 2021). Several strategies have been proposed to turn cold tumors into hot tumors: enhancing inflammation in the TME of cold tumors, inhibiting the peritumoral immunosuppressive state, targeting aberrant tumor vasculature, attenuating tumor-cell-intrinsic pathways, and increasing TILs (Ochoa et al., 2020). Nevertheless, the underlying mechanisms whereby cold tumors are formed have yet to be determined.

N^6^-Methyladenosine (m^6^A) modification, which was first discovered in the 1970s, has gained increasing attention for its important role in eukaryotic epigenetic regulation (Desrosiers et al., 1974; Huang et al., 2020a). Indeed, eukaryotic m^6^A messenger RNA (mRNA) modification is intimately related with almost all cellular and biological processes (Roignant and Soller, 2017). Recently, it was shown that m^6^A RNA modification has a close relationship with the immune response in the TME, suggesting its potential molecular role in the formation of cold tumors and use as a target to improve anticancer immunotherapy (Han D. et al., 2019). However, the researches focus on m^6^A RNA modification in tumor immunology is a novel frontier in cancer research, which not only reveals a new layer of epigenetic regulation in cancer by regulating immune response but can also lead to the development of effective novel therapeutics. In this review, we first describe the TME, cold and hot tumors, and m^6^A RNA modification. Then, we focus on the underlying mechanisms whereby m^6^A RNA modification may be implicated in cold tumor formation. Finally, we discuss the potential clinical implications of m^6^A RNA modification in cancer, and the immunotherapeutic strategies available for its targeting.

## TME in Hot and Cold Tumors

### Hot, Altered, and Cold Tumors

In 1863, Rudolf Virchow first observed that tumor tissues contain leukocytes, indicating an intimate correlation between inflammation and cancer (Balkwill and Mantovani, 2001). Over the past decades, studies involved in elucidating cancer-associated mechanisms have increased our understanding of the complex TME, which is composed of cellular and non-cellular components. The cellular components include fibroblasts and tumor cells, vascular endothelial cells, and immunosuppressive and antitumor immune cells; extracellular matrix (ECM), oxygen, and metabolites constitute the non-cellular components (Binnewies et al., 2018). The composition of the TME explains why traditional chemoradiotherapeutic approaches directly targeting tumor cells are often non-effective. Immunotherapy is an emerging clinical therapeutic approach that focuses on targeting immune cells. It is worth noting that a wide range of tumor patients exhibit resistance to immunotherapy. It is generally accepted that the efficacy of immunotherapeutic approaches and prognosis depend on the density and diversity of immune cells within the tumor site (Fridman et al., 2012). Accordingly, tumors are classified into hot (highly infiltrated) and cold (non-infiltrated) tumors based on the presence and absence of TILs, respectively. Hot tumors appear to have an effective response to anti-CTLA-4, anti-PD-1, and anti-PD-L1 immunotherapies, while cold tumors do not respond to these immunotherapies (Gajewski, 2015). Hot tumors are characterized by high levels of TILs, accumulation of proinflammatory cytokines such as IFN-γ, activation of inhibitory checkpoints (CTLA-4, PD-L1, etc.), genomic instability, presence of immunosuppressive factors such as indoleamine-pyrrole 2,3-dioxygenase 1 (IDO1), and the activation of major histocompatibility complex class I (MHC I). In contrast, cold tumors are characterized by poor lymphocyte infiltration inside the tumor and tumor stroma, absence of PD-L1, low mutational burden, and poor antigen presentation (loss of MHC I, IFN-γ defects, etc.) (Hegde et al., 2016). In 2009, Camus et al. (2009) described another type of tumors known as “altered tumors,” which contain stromal T cells, prevent T-cell infiltration inside of tumors, and present phenotypes that are between those of hot and cold tumors. Altered tumors are characterized by the activation of tumor-cell-intrinsic oncogene pathways such as Wnt/β-catenin and nuclear factor kappa-B (NF-κB); presence of tumor-soluble inhibitory mediators such as vascular endothelial growth factor (VEGF) and transforming growth factor-β (TGF-β); increased levels of immunosuppressive cells such as myeloid-derived suppressor cells (MDSCs), regulatory T cells (Tregs), and tumor-associated macrophages (TAMs); epigenetic changes in the TME; and metabolic competition (hypoxia, overconsumption of glucose, etc.) (Galon and Bruni, 2019). Both cold and altered tumors are derived from tumor-cell-intrinsic immunosuppression and impede effective antitumor immunity. Thus, in order for immunotherapies to have more impact, cold/altered tumors must be turned into hot tumors (Galon and Bruni, 2019; Ochoa et al., 2020).

### Strategies to Turn Cold Tumors Into Hot Tumors

**FIGURE 1:**
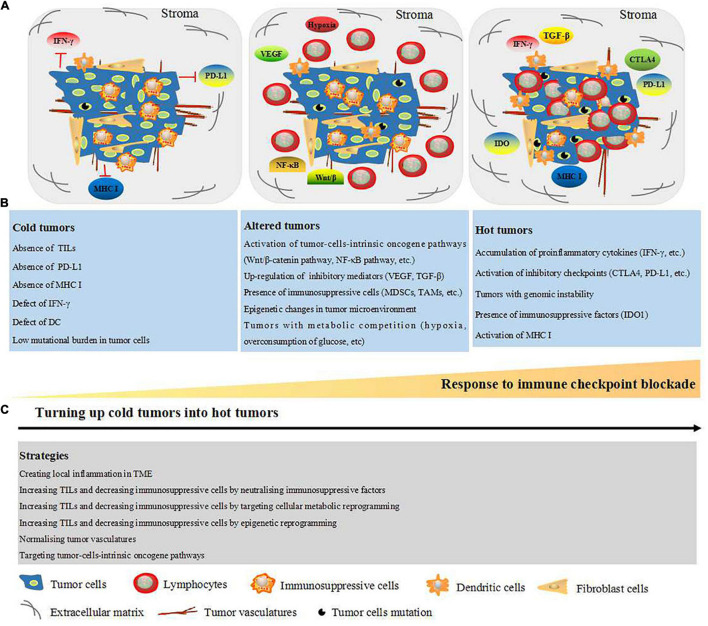
Schematic representation of TME-dependent hot, altered, and cold tumors and strategies to turn up cold tumors into hot tumors. **(A)** TME consist of cellular components: tumor cells, fibroblast cells, DC, immunosuppressive cells [MDSCs, regulatory T cells (TAMs)], and lymphocyte (mainly T cell). Non-cellular components: tumor vasculature, ECM, oxygen, and metabolites. **(B)** Based on the TILs within the tumor site and response to immune checkpoint blockade, the tumors are classified into cold, altered, and cold tumors. Cold tumors are non-effective to immune checkpoint blockade and characterized with absence of TILs, PD-L1, MHC I, IFN-γ, and DC, which are all essential for neoantigen presentation. Furthermore, cold tumors are presented as low mutational burden in tumor cells. Altered tumors are represented with stromal T cells as well as the factors which prevent infiltration of T cells into the tumors, such as activation of tumor-cell-intrinsic oncogene pathways, upregulation of soluble inhibitory mediators (VEGF and TGF-β), and presence of immunosuppressive cells (MDSCs, TAMs, and regulator T cell). Moreover, epigenetic changes and metabolic competition (hypoxia and overconsumption of glucose) in tumor microenvironment are presented in the altered tumors. Hot tumors are represented with high degree of TILs and sensitive to immune checkpoint blockade. Additionally, hot tumors are characterized with accumulation of proinflammatory cytokines (IFN-γ, etc.), inhibitory checkpoints (CTLA-4, PD-L1, etc.), IDO1, MHC I, and genomic instability (high tumors mutation burden). **(C)** Strategies to turn up cold tumors into hot tumors including creating local inflammation in TME, increasing TILs, and decreasing immunosuppressive cells by neutralizing immunosuppressive factors, targeting cellular metabolic reprogramming, targeting epigenetic reprogramming, targeting tumor-cell-intrinsic oncogene pathways, and normalizing tumor vasculature. CTLA-4, cytotoxic T-lymphocyte-associated antigen 4; DC, dendritic cell; ECM, extracellular matrix; IDO1, indoleamine 2,3-dioxygenase 1; IFN-γ, interferon gamma; TME, tumor microenvironment; MDSCs, myeloid derived suppressor cells; MHC I, major histocompatibility complex class I; PD-L1, programmed death-ligand 1; TAMs, tumor-associated macrophage; TGF-β, transforming growth factor-beta; TILs, tumor-infiltrating lymphocytes; VEGF, vascular endothelial growth factor.

Based on the classification into hot, altered, and cold tumors, researchers have explored different strategies to turn cold tumors into hot tumors. For example, the colony-stimulating factor-1 receptor (CSF-1R) is an attractive combination immunotherapeutic agent for tumor treatment by targeting TAMs (Mok et al., 2014; Cannarile et al., 2017; Razak et al., 2020). Furthermore, combined intratumoral interleukin (IL)-12 application with CTLA-4 was shown to lead to glioblastoma eradication through the elevation of CD4^+^ T-cell counts and Treg attenuation (Vom et al., 2013). As our understanding of cold and hot tumors expanded, strategies to turn cold tumors into hot tumors have been reported including creating local inflammation in the TME, increasing the levels of TILs, and decreasing levels of immunosuppressive cells by neutralizing immunosuppressive factors, targeting cellular metabolic and epigenetic reprogramming, normalizing tumor vasculature, and targeting tumor-cell-intrinsic oncogene pathways (Duan et al., 2020; Ochoa et al., 2020). An overview of the characteristics of hot, altered, and cold tumors as well as the strategies to turn cold tumors into hot ones is presented in Figure 1.

## m^6^A RNA Modification

### Discovery and Characteristics of m^6^A RNA Modification

**FIGURE 2:**
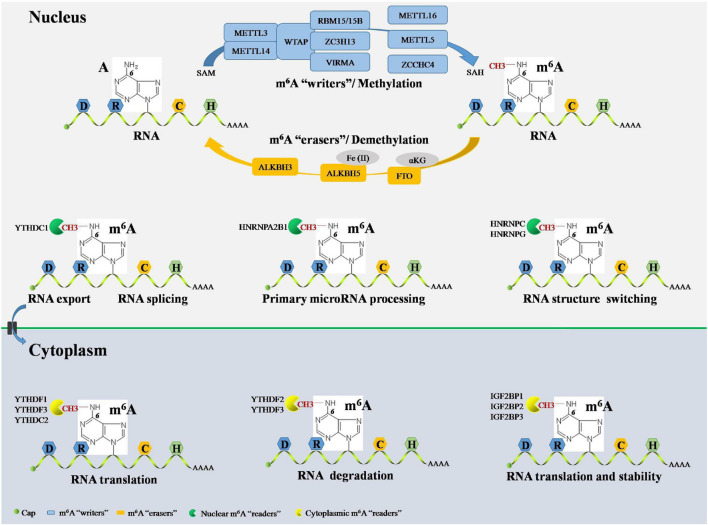
Overview RNA m^6^A modification by its “writers,” “erasers,” and “readers.” The RNA m^6^A have a consensus sequence DRACH sites and methylated A at the N^6^ position. In nucleus, m^6^A methylation in RNA can be installed by m^6^A writers complex, including METTL3, METTL14, WTAP, RBM15, RBM15B, and ZC3H13. RNA m^6^A methylation also can be installed by several writers independently, including METTL16, METTL5, and ZCCHC4. The initiate of RNA m^6^A modification is dependent on methyl donor SAM and terminate in SAH production. The RNA m^6^A can be reversibly and dynamically removed by m^6^A erasers in nucleus composed of FTO, ALKBH5, and ALKBH3. FTO-mediated RNA m^6^A demethylation is αKG dependent, and ALKBH5-mediated RNA m^6^A demethylation is Fe(II) dependent. The RNA m^6^A can be recognized by m^6^A readers both in the nucleus and cytoplasm. Cytoplasmic m^6^A readers include YTHDF1, YTHDF2, YTHDF3, IGF2BP1, IGF2BP2, IGF2BP3, and YTHDC2. YTHDF1 and YTHDC2 promote RNA translation. YTHDF2 facilitates RNA degradation. YTHDF3 cooperates with YTHDF1 to promote RNA translation and synergy with YTHDF2 to facilitate RNA degradation. IGF2BP1, IGF2BP2, and IGF2BP3 are essential for promoting the stability and translation of RNA. Nuclear m^6^A readers consist of YTHDC1, HNRNPA2B1, HNRNPC, and HNRNPG. YTHDC1 contributes to RNA splicing and RNA export from nucleus to cytoplasm. HNRNPA2B1 causes primary microRNA processing. HNRNPC and HNRNPG RNA end with structure switching. m^6^A, N^6^-methyladenosine; A, adenosine; C, cytidine; METTL, methyltransferase-like; WTAP, Wilms’ tumor 1-associated protein; RBM, RNA-binding motif; ZC3H13, zinc finger CCCH-type containing 13; ZCCHC4, zinc finger CCHC-type containing 4; SAM, *S*-adenosylmethionine; SAH, *S*-adenosyl homocysteine; FTO, fat mass and obesity-associated protein; ALKBH, ALKB homolog; αKG, α-ketoglutarate; YTHDF, YT521-B homology domain-containing family; YTHDC, YT521-B homology domain-containing protein; IGF2BP, insulin-like growth factor-2 mRNA-binding protein; HNRNP, heterogeneous nuclear ribonucleoprotein.

Epigenetic events are implicated in almost all major bioprocesses. These epigenetic events, which consist of DNA methylation, histone modification, and RNA-mediated processes, are reversible and dynamic chemical modifications (Ling and Ronn, 2019). These modifications are cooperatively interpreted by a multitude of guiding enzymes that can be classified into “writer,” “eraser,” and “reader” proteins. Disruption of any of these proteins contributes to disease development, including cancer (Dawson, 2017). DNA methylation and histone modification are essential for controlling chromatin remodeling and gene expression epigenetically. Nevertheless, the field of RNA-mediated processes has not moved forward very much (Deng et al., 2018; Jung et al., 2020). There is still a lot to uncover in terms of RNA-mediated processes, their regulation, and effects, etc., but more than 160 chemical RNA modifications have been identified since the 1950s, advancing our understanding of the biogenesis and function of RNA (Saletore et al., 2012). m^6^A, the methylation of adenosine (A) at the N^6^ position, was the first identified RNA modification and has been defined as the most widespread internal chemical modification in eukaryotic mRNA. Furthermore, m^6^A has also been identified in non-coding RNAs, such as ribosomal (rRNAs), small nuclear (snRNAs), small nucleolar (snoRNAs), micro- (microRNAs), long non-coding (lncRNAs), and circular (circRNAs) RNAs (Dominissini et al., 2012). Next-generation sequencing (NGS) studies have shown that m^6^A RNA modification sites in mRNA, microRNAs, lncRNAs, and circRNAs are non-randomly distributed but have the DRACH consensus sequence (D = G/A/U; R = G/A; H = A/C/U; G/C/U: guanosine/cytidine/uridine) and are highly enriched in the coding sequence, 3′-untranslated region (3′-UTR), and around stop codons (Meyer et al., 2012). Notably, the development of NGS-based approaches for m^6^A sequencing promises to delineate the landscape of the m^6^A epitranscriptome in various cellular contexts (Garcia-Campos et al., 2019; Huang et al., 2020b). In line with DNA methylation and histone modification, m^6^A RNA modification is a reversible and dynamic process that can be installed, removed, and recognized by its writers, erasers, and readers, respectively (Wang et al., 2020d; Figure 2).

### m^6^A Writers

m^6^A writers install m^6^A through a methyltransferase complex (MTC) composed of several components. Methyltransferase-like 3 (METTL3), METTL14, and Wilms’ tumor 1-associated protein (WTAP) are core components of the m^6^A MTC (Bokar et al., 1997). METTL3 is the only catalytic subunit, which installs m^6^A by binding to the methyl donor, *S*-adenosylmethionine (SAM), and transferring the methyl groups to adenine in the RNA molecule, producing *S*-adenosyl homocysteine (SAH). METTL3 and METTL14 are co-localized in nuclear speckles and form METTL3-METTL14 heterodimer complexes in a 1:1 ratio. METTL14 also contains the catalytic donor; however, METTL14 itself is not a catalytic subunit but maintains METTL3 conformation and identifies catalytic substrates (Wang P. et al., 2016; Wang X. et al., 2016). Moreover, METTL14 cooperates with the histone mark, histone H3 lysine 36 trimethylation (H3K36me3), to carry out m^6^A RNA methylation, suggesting a co-transcriptional mechanism underlying histone modification and RNA methylation in mammalian transcriptomes (Huang et al., 2019). WTAP does not have catalytic function but facilitates m^6^A deposition through recruitment of METTL3-METTL14 heterodimer complexes as well as localization to nuclear speckles (Ping et al., 2014). RNA-binding motif protein 15 (RBM15) and RBM15B, which have no catalytic function, interacts with METTL3 and WTAP and assists these two core components to reach their target RNA sites for m^6^A RNA modification in nuclear speckles (Knuckles et al., 2018). Zinc finger CCCH-type containing 13 (ZC3H13) controls the MTC by binding to WTAP and is required for the nuclear localization of the ZC3H13-WTAP-Virilizer-Hakai complex, which is essential for facilitating m^6^A methylation and mouse embryonic stem cell pluripotency (Wen et al., 2018). Vir-like m^6^A methyltransferase associated (VIRMA), also called KIAA1429, mediates preferential m6A mRNA methylation in the 3′-UTR and near stop codon (Yue et al., 2018). Furthermore, the MTC contains other components, such as METTL16 and METTL5. METTL16 has been suggested to function alone in catalyzing m^6^A modification on the U6 snRNA (Warda et al., 2017), whereas METTL5 acts as an independent RNA methyltransferase and is required for 18S rRNA m^6^A modification (Leismann et al., 2020). Moreover, zinc finger CCHC-type containing 4 (ZCCHC4) was identified as an RNA methyltransferase in 2019 and is essential for the independent methylation of 28S rRNA (Ma et al., 2019).

### m^6^A Erasers

m^6^A RNA modification can be removed by a handful of specific demethylases known as erasers. The fat mass and obesity-associated protein (FTO) was identified as the first m^6^A demethylase in 2011 (Jia et al., 2011). FTO is an α-ketoglutarate (αKG)-dependent demethylase located in both the cell nucleus and cytoplasm (Gulati et al., 2014). FTO first oxidizes m^6^A to form N^6^-hydroxymethyladenosine (hm^6^A). Then, hm^6^A is converted to N^6^-formyladenosine (f^6^A). Lastly, f^6^A is converted to adenosine, thus removing the m^6^A RNA modification in the nucleus (Wang et al., 2020d). Furthermore, FTO also demethylates N^6^,2′-*O*-dimethyladenosine (m^6^A_m_) in snRNA and N^1^-methyladenosine (m^1^A) in tRNA in the nucleus (Wei et al., 2018). It is worth mentioning that FTO can mediate mRNA and cap m^6^A_m_ demethylation as well as tRNA m^1^A demethylation in the cytoplasm (Wei et al., 2018). Moreover, ALKB homolog 5 (ALKBH5) is another vital m^6^A eraser, which is Fe(II) dependent, locates in the nucleus, and seems to be an m^6^A-specific demethylase involved in m^6^A RNA modification (Zheng et al., 2013). Moreover, Ueda et al. (2017) recently identified ALKBH3, an m^6^A eraser suggested to be present in both, in the cytoplasm and nucleus, promoting the demethylation of target mammalian tRNA.

### m^6^A Readers

The reversible processes of m^6^A RNA installation and removal occur through the alteration of the RNA structure. RNA-mediated biological functions are also regulated by m^6^A-binding proteins, which are called m^6^A readers (Li A. et al., 2017). On the one hand, cytoplasmic mRNA is decoded in the ribosome to produce a protein. On the other hand, messenger ribonucleoprotein (mRNP) foci are essential for the storage or degradation of cytoplasmic RNA. The YT521-B homology (YTH) domain-containing proteins (YTHDFs) and insulin-like growth factor-2 mRNA-binding proteins (IGF2BPs) play crucial roles in RNA-mediated biological functions by binding to m^6^A domains in the cytoplasm. YTHDFs include YTHDF1, YTHDF2, and YTHDF3. YTHDF1 selectively binds to m^6^A and recruits translation initiation factors, including the eukaryotic translation initiation factors (eIFs) 3/4E/4G, poly(A) binding protein (PABP), and 40S ribosomal subunit, to magnify RNA translation (Wang et al., 2015). The first identified m^6^A reader was YTHDF2, which recognizes m^6^A-modified RNA degradation sites *via* its C-terminal region and recruits the carbon catabolite repressor 4-negative on TATA (CCR4-NOT) deadenylase complex through its N-terminal region (Du et al., 2016; Zhang C. et al., 2020). YTHDF3 has overlapping roles in RNA fate through augmenting RNA translation in cooperation with YTHDF1 and promoting RNA degradation *via* synergy with YTHDF2 (Li A. et al., 2017; Shi et al., 2017). Cytoplasmic IGF2BPs, including IGF2BP1, IGF2BP2, and IGF2BP3, bind directly to m^6^A-modified RNA through its K homology domains and promote the stability and translation of RNA (Kataoka, 2019). Cytoplasmic YTH domain-containing protein 2 (YTHDC2) is another m^6^A reader that can recognize m^6^A and bind to meiosis-specific coiled-coil domain (MEIOC) and 5′-3′exoribonuclease 1, further increasing m^6^A-modified RNA translation (Hsu et al., 2017). Notably, m^6^A readers can also bind m^6^A in the nucleus. For example, YTHDC1 promotes exon inclusion in RNA by amplifying serine- and arginine-rich splicing factor 3 (SRSF3) or blocking serine- and arginine-rich splicing factor 10 (SRSF10) in the nucleus (Xiao et al., 2016). Furthermore, YTHDC1 plays a role in facilitating m^6^A-methylated RNA export from the nucleus to the cytoplasm (Roundtree et al., 2017). Additionally, heterogeneous nuclear ribonucleoproteins (hnRNPs), including HNRNPA2B1, HNRNPC, and HNRNPG, recognize m^6^A and act as “m^6^A switches” that accelerate RNA and primary microRNA processing by changing the RNA structure (Alarcon et al., 2015; Liu et al., 2015; Zhou et al., 2019).

In summary, studies have shown that m^6^A RNA modifications are implicated in a wide range of biological processes. Nevertheless, structural and biochemical data on m^6^A writers, erasers, and readers need to be further verified, and the detailed mechanisms regulated by these proteins remain undetermined. It is reasonable to believe that there are more m^6^A writer, eraser, and reader components, and that the mechanism underlying these protein-mediated RNA modifications will be elucidated with the development of quantification and sequencing methodologies (Bodi and Fray, 2017; Chen et al., 2019). A summary of the currently known m^6^A writers, erasers, and readers is presented in Table 1.

**TABLE 1:** The locations and mechanisms of RNA m6A modification regulators.

Categories	Regulators	Locations	Mechanisms	References
m^6^A “writers”	METTL3	Nucleus	The only catalytic subunit that installs m^6^A methylation by binding to SAM and producing SAH	Wang P. et al., 2016; Wang X. et al., 2016
	METTL14	Nucleus	Forming METTL3-METTL14 heterodimer and steadies METTL3 conformation and identifies catalytic substrates; cooperates with the H3K36me3 to install RNA m^6^A methylation	Wang P. et al., 2016; Wang X. et al., 2016; Huang et al., 2019
	WTAP	Nucleus	Facilitating m^6^A deposition by recruiting METTL3-METTL14 heterodimer complex localization to nuclear speckles	Ping et al., 2014
	RBM15/15B	Nucleus	Assisting METTL3 and WTAP to their target RNA sites for RNA m^6^A modification in nuclear speckles	Knuckles et al., 2018
	ZC3H13	Nucleus	Binding to WTAP and induces the nuclear localization of ZC3H13-WTAP-Virilizer-Hakai complex	Wen et al., 2018
	VIRMA	Nucleus	Guiding region-selective mRNA m^6^A modification in 3′-UTR and near stop codon	Yue et al., 2018
	METTL16	Nucleus	Functioning alone in catalyzing m^6^A modification on U6 snRNA	Warda et al., 2017
	METTL5	Nucleus	Acting alone in catalyzing 18S rRNA m^6^A modification	Leismann et al., 2020
	ZCCHC4	Nucleus	Functioning alone in catalyzing 28S rRNA m^6^A modification	Ma et al., 2019
m^6^A “erasers”	FTO	Nucleus	Promoting m^6^A modification in RNA removed dependent on αKG; inducing RNA demethylation of m^6^A_m_ in snRNA and m^1^A in tRNA	Gulati et al., 2014; Wei et al., 2018
	FTO	Cytoplasm	Promoting m^6^A_m_ demethylation as well as tRNA m^1^A demethylation	Wei et al., 2018
	ALKBH5	Nucleus	Inducing m^6^A demethylation dependent on Fe (II)	Zheng et al., 2013
	ALKBH3	Nucleus/cytoplasm	Promoting demethylation of target mammalian tRNA	Ueda et al., 2017
m^6^A “readers”	YTHDF1	Cytoplasm	Recruiting eIF3/4E/4G, PABP, and 40S ribosomal subunit to magnify RNA translation	Wang et al., 2015
	YTHDF2	Cytoplasm	Recognizing m^6^A-modified RNA degradation sites by its C-terminal region, and recruiting carbon CCR4-NOT deadenylase complex by its N-terminal region	Du et al., 2016; Zhang C. et al., 2020
	YTHDF3	Cytoplasm	Increasing RNA translation in cooperation with YTHDF1 and promoting RNA degradation by synergy with YTHDF2	Li A. et al., 2017; Shi et al., 2017
	IGF2BP1/ 2/ 3	Cytoplasm	Promoting the stability and translation of RNA by binding to m^6^A-modified RNA through its K homology domains	Kataoka, 2019
	YTHDC2	Cytoplasm	Increasing m^6^A-modified RNA translation by binding to MEIOC and 5′-3′exoribonuclease 1	Hsu et al., 2017
	YTHDC1	Nucleus	Promoting RNA splicing and facilitating m^6^A-methylated RNA exportation from nucleus to cytoplasm	Xiao et al., 2016; Roundtree et al., 2017
	HNRNPA2B1	Nucleus	Acting as “m^6^A switch” to accelerate primary microRNA processing	Alarcon et al., 2015
	HNRNPC/ G	Nucleus	Acting as “m^6^A switch” to change the structure of RNA	Liu et al., 2015; Zhou et al., 2019

*Abbreviations: 3′-UTR: 3′ untranslated region; αKG: α-ketoglutarate; ALKBH: ALKB homolog; CCR4-NOT: carbon catabolite repressor 4-negative on TATA; eIF: eukaryotic translation initiation factor; FTO: fat mass and obesity-associated protein; H3K36me3: histone H3 lysine 36 trimethylation; HNRNP: heterogeneous nuclear ribonucleoprotein; IGF2BP: insulin-like growth factor-2 mRNA-binding protein; m^1^A: N^1^-methyladenosine; m^6^A: N^6^-methyladenosine; m^6^A_m_: N^6^, 2′-O-dimethyladenosine; METTL: methyltransferase-like; MEIOC: meiosis-specific coiled-coil domain; PABP: poly(A) binding protein; PD-1: programmed death receptor 1; RBM: RNA-binding motif; rRNA: ribosomal RNAs; SAH: S-adenosyl homocysteine; SAM: S-adenosylmethionine; SnRNA: small nuclear RNAs; tRNA: transfer RNA; VIRMA: vir-like m^6^A methyltransferase associated; WTAP: Wilms’ tumor 1-associated protein; YTHDC: YTH domain-containing protein; YTHDF: YTH domain-containing family; ZC3H13: zinc finger CCCH-type containing 13; ZCCHC4: zinc finger CCHC-type containing 4.*

## Aberrant m^6^A RNA Modification in Cold Tumors

With the breakthrough in the field of m^6^A RNA modification research during the past decade, reversible and dynamic m^6^A RNA modifications have been reported in almost all normal physiological processes. Comprehensive studies have shown that the regulators of m^6^A RNA modification are systematically implicated in the formation of complex TMEs, affecting the immune microenvironment, tumor mutational burden, neoantigen load, immunotherapy response, and even survival (Zhang B. et al., 2020; He et al., 2021; Wu et al., 2021; Xu et al., 2021b). Recently, studies have demonstrated that the aberration/imbalance of m^6^A RNA modification has a close relationship with immune disorders in cancer (Li H. B. et al., 2017; Su et al., 2020; Kim et al., 2021). These findings suggest a role of m^6^A RNA modification in cold tumors.

### m^6^A RNA Modification and Immunity in the TME

In colorectal cancers with low mutational burden, which are resistant to immunotherapy, depletion of METTL3 and METTL14 increases the expression of CD8^+^ T cells and the secretion of IFN-γ *via* the m^6^A reader YTHDF2 (Wang et al., 2020b). Another study showed that tumors with decreased levels of METTL3 have increased DC infiltration, MHC expression, and levels of costimulatory and adhesion molecules in the TME (Shen et al., 2021a). On the contrary, loss of METTL3 has also been shown to promote tumor growth and metastasis. For example, METTL3-deficient mice show increased immunosuppressive cell (TAMs, Tregs) infiltration into tumors (Yin et al., 2021). Yao et al. (2021) showed that METTL3 is responsible for the expression of T follicular helper cells, which are specialized effector CD4^+^ T cells. Loss of METTL3 results in inactivation of T follicular helper cell differentiation by promoting the decay of T follicular helper cell signature genes, including Tcf7 transcripts. Using CRISPR-Cas9 screening, Tong et al. (2021) demonstrated that loss of METTL3 leads to the removal of m^6^A RNA modification on Irakm IL-1 receptor-associated kinase 3 (IRAK3) mRNA, slowing down its degradation and ultimately attenuating toll-like receptor 4 (TLR4) signaling-mediated macrophage activation. Particularly, the authors suggested that METTL3 augments the tumoricidal ability of macrophages by promoting the polarization bias of TAMs toward the M1 macrophage phenotype and rescuing infiltrating CD4^+^ and CD8^+^ T cells (Tong et al., 2021). Recently, mechanistic investigations found a positive role of ALKBH5 in Tregs and MDSCs by targeting Mct4/Slc16a3. Notably, low levels of ALKBH5 in clinical settings are correlated with low Treg cell numbers (Li et al., 2020c). However, another study by Tang et al. (2020) showed that deletion of ALKBH5 decreases the infiltration of CD8^+^ T cells in pancreatic adenocarcinoma.

Lysosomal proteases are responsible for antigen degradation in DCs (Cebrian et al., 2011). In a study by Han D. et al. (2019), YTHDF1 was shown to have a negative correlation with CD8^+^ T-cell infiltration in colon cancer patients. Mechanistically, YTHDF1 in DCs can recognize lysosomal proteases, leading to the inactivation of cross-presentation. Loss of YTHDF1 promotes DC-mediated cross-presentation of tumor antigens and cross-priming of CD8^+^ T cells *in vitro* and *in vivo* (Han D. et al., 2019). Additionally, other m^6^A RNA modification regulators have also been found to have a close relationship with immune cells in tumors. For instance, the expression of METTL14 and ZC3H13 is positively correlated with infiltrating levels of CD4^+^ T cells, CD8^+^ T cells, and DCs, but negatively correlated with those of Tregs in breast cancer (Gong et al., 2020). In head and neck squamous cell carcinoma, low expression of YTHDC2 is positively correlated with the low levels of B cells, CD8^+^ T cells, CD4^+^ T cells, neutrophils, and infiltrating DCs (Li et al., 2020d). IFN-γ is the main proinflammatory cytokine produced by cytotoxic T cells, enhancing antigen presentation to cytotoxic T cells by facilitating MHC I and immunoproteasome expression in tumor cells (Cheon et al., 2014). YTHDF2 is responsible for RNA-binding motif 4 (RBM4)-mediated suppression of IFN-γ-induced M1 macrophage polarization and glycolysis (Huangfu et al., 2020). In a recent study by Shen et al. (2021a), downregulation of METTL3 was shown to contribute to increasing the levels of MHC molecules (Shen et al., 2021a). More recently, the levels of YTHDC2, HNRNPC, and VIRMA were suggested to be negatively correlated, whereas WTAP was positively correlated, with MHC molecules in endometrial cancer (Zhao et al., 2021). A comprehensive study showed that a low risk score of m^6^A signature is significantly correlated with a high expression of immune cell checkpoint molecules, such as PD-1, PD-L1, and CTLA-4 (Mo et al., 2020). Nevertheless, the mechanisms whereby m^6^A RNA modification regulators exert their action in immune cells of the TME remain unclear.

### m^6^A RNA Modification and Tumor-Cell-Intrinsic Pathways

Several studies have shown that METTL3 acts as an oncogenic regulator by activating tumor-cell-intrinsic pathways in tumors. For example, in hepatoblastoma, upregulation of METTL3 promotes the proliferation, migration, and invasion of hepatoblastoma cells by activating the Wnt/β-catenin signaling pathway (Liu et al., 2019; Cui et al., 2020). In colorectal cancer, METTL3 promotes tumor metastasis, stemness, and chemoresistance through activation of MAPK and Wnt/β-catenin signaling (Peng et al., 2019; Liu et al., 2021b). Furthermore, METTL3 facilitates the proliferation and invasion of esophageal cancer cells *via* activation of Wnt/β-catenin and AKT signaling (Hou et al., 2020). In contrast, Yin et al. (2021) recently showed that ablation of METTL3 orchestrates tumor growth and metastasis by facilitating ERK-NF-κB/STAT3 signaling. Liu et al. (2018) showed that METTL14 mutation and loss of METTL3 expression contribute to increased proliferation and tumorigenicity of endometrial cancer cells by activating AKT signaling. Moreover, METTL3 knockdown in a multiplicity of tumor cell lines leads to the activation of PI3K/AKT/mTOR signaling (Zhao et al., 2020). Wang Y. et al. (2021) indicated that METTL14 may be a favorable prognostic factor for clear cell renal cell carcinoma (ccRCC). Mechanistically, loss of METTL14 increases gastric cancer cell proliferation and invasiveness by promoting the activation of Wnt and PI3K/AKT signaling. In contrast, knockdown of FTO restricts the activation of Wnt and PI3K/AKT signaling (Zhang et al., 2019). Recently, Liu et al. (2021a) showed that METTL3 and METTL14 are required for senescence-associated secretory phenotype (SASP)-mediated tumor-promoting and immune-surveillance functions of senescent cells through the activation of NF-κB signaling. Frizzled proteins are key Wnt receptors whose activation contributes to the stabilization of cytoplasmic β-catenin (MacDonald and He, 2012). The activity of FTO and ALKBH5 lead to PARP inhibitor resistance in BRCA-deficient epithelial ovarian cancer (EOC) cells by upregulating the Wnt/β-catenin pathway through stabilization of Frizzled 10 protein (Fukumoto et al., 2019). YTHDF1 has been shown to promote stemness, tumor cell proliferation, and metastasis by activating the Wnt/β-catenin pathway through the stabilization of Frizzled 5 and 7 (Bai et al., 2019; Han et al., 2020; Liu et al., 2020; Pi et al., 2021).

### m^6^A RNA Modification and Soluble Inhibitory Mediators in the TME

As mentioned earlier, altered tumors are characterized by the presence of tumor angiogenesis. METTL3 has been shown to facilitate miR-143-3p biogenesis, promoting the brain metastasis in lung cancer patient samples through the miR-143-3p/Vasohibin/VEGFA axis (Wang et al., 2019). In line therewith, Wang G. et al. (2021) showed that METTL3 is responsible for the activation of tyrosine kinase endothelial (TEK)-VEGFA-mediated tumor progression and angiogenesis in bladder cancer. In colon cancer, the m^6^A RNA modification reader, IGF2BP3, can bind to the VEGF mRNA to promote its expression and stability. Thus, loss of IGF2BP3 restricts angiogenesis by inhibiting VEGF (Yang et al., 2020). Upregulation of TGF-β in the TME also contributes to altered tumor formation by suppressing T-cell proliferation and stimulating Treg development (Chen and Ten Dijke, 2016). In TGF-β-induced epithelial-mesenchymal transition (EMT) of lung cancer cell lines, the level of METTL3 was found to be upregulated. Loss of METTL3 attenuates TGF-β-induced morphological conversion of lung cancer cells, their cell migration potential, and EMT progression (Wanna-Udom et al., 2020). Mechanistic investigations found that METTL3 increases TGF-β1 mRNA decay and impairs TGF-β1 translation progress. Furthermore, ablation of METTL3 disrupts the autocrine action of TGF-β1 by interrupting TGF-β1 dimer formation and TGF-β1-induced EMT in cancer cells (Li et al., 2020a). Importantly, the level of VEGFA and content of TGF-β1 in the TME are decreased in ALKBH5-deficient melanoma cells (Li et al., 2020c).

### m^6^A RNA Modification and Metabolic Competition in the TME

Recently, m^6^A RNA modification was recognized to be responsible for metabolic competition-mediated tumorigenesis. Cancer cells with metabolic competition contribute to tumorigenesis through inhibiting T-cell responses and increasing T-cell depletion (Kedia-Mehta and Finlay, 2019). Upregulation of ALKBH5 was shown to contribute to breast cancer initiation by attenuating NANOG mRNA methylation and thereby increasing NANOG expression under hypoxia (Zhang et al., 2016). FTO was found upregulated in tumor suppressor von Hippel-Lindau (VHL)-deficient ccRCC. Mechanistically, FTO increases metabolic reprogramming and survival of VHL-deficient ccRCC cells by targeting SLC1A5 in a hypoxia-inducible factor (HIF)-independent way (Xiao et al., 2020). Furthermore, Niu et al. (2021) recently showed that the posttranscriptional regulation of the abnormal expression of aldolase A (ALDOA) under hypoxia was positively modulated by FTO-mediated m^6^A RNA modification in a YTHDF2-dependent manner in liver cancer cells, and hypoxia-mediated high level of ALDOA contributed to liver cancer development by promoting glycolysis metabolism and its terminal product lactate expression. Additionally, FTO promotes tumor cell glycolysis by activating PI3K/AKT signaling or in a YTHDF2-dependent manner (Liu et al., 2017; Liu et al., 2021d). In addition, upregulation of METTL3 in gastric cancer promotes tumor angiogenesis and glycolysis by promoting IGF2BP3-dependent hepatoma-derived growth factor (HDGF) mRNA stability, which is essential for increasing in glycolysis by activating GLUT4 and ENO2 in gastric cancer cells (Wang et al., 2020c).

### m^6^A RNA Modification and Tumor Mutational Burden

Tumors with high mutational burden carry neoantigens that are sensitive to immune cells and immune checkpoint blockade (Samstein et al., 2019). Recently, numerous systematic and comprehensive studies have suggested a close relationship between m^6^A RNA modification and mutational burden. m^6^A RNA modification patterns are quantified as m^6^Ascore by a specific procedure (Zeng et al., 2019). Zhang B. et al. (2020) comprehensively investigated the m^6^A RNA modification patterns of 1,938 gastric cancer samples based on 21 m^6^A regulators and systematically analyzed the correlation between the m^6^Ascore and TME cell-infiltrating characteristics. They found that a low m^6^Ascore is markedly correlated with increased mutational burden and activation of immunity and correlated with increased neoantigen load and enhanced response to anti-PD-1/L1 treatment (Zhang B. et al., 2020). Another study showed a wide range of FTO, RBM15, and YTHDF1 inter-group expression differences between high- and low- tumor mutational burden cancer tissues (Liu et al., 2021c). Consistently, a recent study indicated that there is a positive correlation between the m^6^A signature and tumor mutational burden scores in 16 cancer types (Shen et al., 2021b). Furthermore, in colon cancer patients, a low m^6^Ascore is associated with high tumor mutational burden, PD-L1 expression, and mutation rates in significantly mutated genes (Chong et al., 2021). It is also noteworthy that colorectal cancers with low mutational burden were suggested to be resistant to anti-PD-1 immunotherapy through the inhibition of IFN-γ-mediated CD8^+^ T-cell secretion by METTL3 and METTL14 (Wang et al., 2020b). Nevertheless, the mechanisms whereby m^6^A RNA modification regulates the tumor mutational burden require further investigation.

**FIGURE 3:**
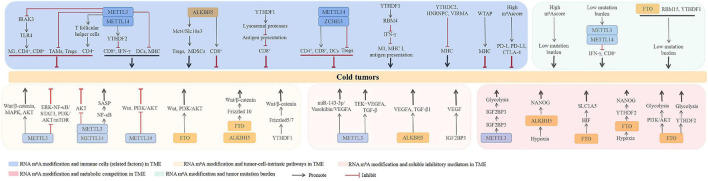
The potential roles of RNA m^6^A modification in cold tumors. The mechanisms underlying RNA m^6^A modification-mediated cold tumors include regulating the immune cells in TME, targeting tumor-cell-intrinsic pathways, facilitating soluble inhibitory mediators in TME, increasing metabolic competition in TME, and affecting tumor mutation burden. ALKBH5, ALKB homolog 5; CTLA-4, cytotoxic T-cell lymphocyte-associated protein 4; DCs, dendritic cells; FTO, fat mass and obesity-associated protein; HNRNP, heterogeneous nuclear ribonucleoprotein; M1, M1 macrophages; MDSCs, myeloid-derived suppressor cells; METTL, methyltransferase-like; MHC, major histocompatibility complex class; NF-κB, nuclear factor kappa-B; HIF, hypoxia-inducible factor; IFN-γ, interferon γ; IRAK3, IL-1 receptor-associated kinase 3; IGF2BP, insulin-like growth factor-2 mRNA-binding protein; PD-1, programmed death receptor 1; PD-L1, programmed death receptor ligand 1; RBM, RNA-binding motif; SASP, senescence-associated secretory phenotype; TAMs, tumor-associated macrophages; TGF-β, transforming growth factor-β; TLR4, toll-like receptors 4; Tregs, regulatory T cells; VEGF, vascular endothelial growth factor; VIRMA, vir-like m^6^A methyltransferase associated; WTAP, Wilms’ tumor 1-associated protein; YTHDC2, YT521-B homology domain-containing protein 2; YTHDFs, YT521-B homology domain-containing family; ZC3H13: zinc finger CCCH-type containing 13.

Collectively, the mechanisms underlying m^6^A RNA modification-mediated cold tumor formation include immune cell regulation in the TME, targeting of tumor-cell-intrinsic pathways, facilitation of soluble inhibitory mediators in the TME, increase of metabolic competition in the TME, and effect on tumor mutational burden. Notably, several specific m^6^A regulators play dual roles in cold tumor formation, such as METTL3, METTL14, and YTHDF1, suggesting the exact role m^6^A RNA modification-mediated cold tumor formation is tumor-type dependent. Furthermore, the abnormal expression of m^6^A regulators contribute to cold tumor formation is not through one mechanism alone, they always play roles in cold tumor formation by several mechanisms. For example, METTL3 is involved in cold tumor formation *via* regulating immune cell expression, targeting of tumor-cell-intrinsic pathways, facilitating soluble inhibitory mediators, increasing metabolic competition, and affecting tumor mutational burden together, which indicated the extensive role of m^6^A RNA modification in cold tumor formation. In addition, some different m^6^A regulators are implicated in cold tumor formation by the same mechanism, such as METTL3 and ALKBH5, they both lead to cold tumor formation through VEGFA expression, indicating they may play a role in the cold tumor formation synergistically, which needs to be validated in the future. The studies involved in m^6^A RNA modification in cold tumor are just getting started; the related mechanism is still unclear and needs to be illustrated in the future. An overview of the uncovered mechanisms till now is presented in Figure 3.

## Potential Clinical Implications of m^6^A RNA Modification in Cancers

Considering the widespread role of m^6^A RNA modification in tumorigenesis, it is reasonable to assume that the expression of m^6^A writers, erasers, and readers might be used as diagnostic or prognostic biomarkers for cancer patients. Recent studies using Kaplan-Meier analysis and receiver operating characteristic curve (ROC) have illustrated that METTL3 has potential clinical implications in cancer. For instance, compared with adjacent non-tumor tissues, METTL3 expression is upregulated in hepatoblastomas. High METTL3 levels are associated with continual recurrence and poor prognosis of hepatoblastoma patients, suggesting that METTL3 could be used as a potential diagnostic and prognostic biomarker for hepatoblastoma patients (Liu et al., 2019; Cui et al., 2020). Furthermore, in bladder cancer, gastric cancer, and colorectal cancer, increased expression of METTL3 correlated with poor prognosis (Han J. et al., 2019; Peng et al., 2019; Wang et al., 2020c). Since METTL3 plays overlapping roles in tumors (Zheng et al., 2019), its high expression was shown to be positively correlated with better survival in colorectal cancer (Deng et al., 2019). Compared with normal samples, the expression of METTL14 and ZC3H13 is decreased in invasive breast cancer stroma, invasive ductal breast cancer stroma, invasive mixed breast cancer, and ductal carcinoma *in situ*. These low levels of METTL14 and ZC3H13 are negatively correlated with overall survival (OS) and progression-free survival (PFS) in luminal type A, luminal type B, human epidermal growth factor receptor 2 (HER2)-enriched type, and triple-negative-type breast cancer, indicating that the reduced expression of METTL14 and ZC3H13 leads to poor prognosis in breast cancer patients (Gong et al., 2020). Additionally, overexpression of ALKBH5 is correlated with poor prognosis in acute myeloid leukemia patients (Shen et al., 2020). Upregulation of YTHDF1 is intimately associated with poor OS in hepatocellular carcinoma (HCC) and gastric cancer patients (Liu et al., 2020; Pi et al., 2021). YTHDF2 is significantly overexpressed in hepatoblastoma and HCC when compared with their adjacent non-cancerous tissues, and overexpression of YTHDF2 is closely connected with poor prognostic clinical outcomes (Cui et al., 2020; Shao et al., 2020). Recently, Li et al. (2020d) showed that head and neck squamous cell carcinoma patients with lower YTHDC2 levels have poorer OS and PFS than those with higher expression. Like METTL3, FTO also plays pro- and antitumor roles in cancer (Wang et al., 2020a). Cui et al. (2020) found that the upregulation of FTO in hepatoblastoma patients is correlated with poor clinical outcomes. However, Zhuang et al. (2019) suggested that low FTO expression is correlated with poor prognosis in endometrial cancer, lung cancer, rectum adenocarcinoma, and pancreatic cancer.

Furthermore, a genome metacohort analysis showed that low FTO and METTL14 levels and high METTL3, HNRNPA2B1, and YTHDF3 levels are correlated with poor prognosis in osteosarcoma patients (Li et al., 2020b). In endometrial cancer patients, higher HNRNPC, YTHDC2, WTAP, VIRMA, IGF2BP3, and HNRNPA2B1 expression is closely associated with worse outcomes and advanced stage (Zhao et al., 2021). Furthermore, high ALKBH5 levels in colon cancer indicates poor prognosis (Huang et al., 2021). In addition, numerous studies used the m^6^Ascore to investigate the potential clinical implications of m^6^A RNA modification patterns in cancer (Zhang B. et al., 2020; Du et al., 2021; Shen et al., 2021a; Xu et al., 2021b). For example, Zhang C. et al. (2020) indicated that the m^6^Ascore can act as an independent prognostic biomarker in gastric cancer. In HCC patients, the OS of the low m^6^Ascore group was better than that of the high m^6^Ascore group (Shen et al., 2021a). Importantly, the OS of low-grade glioma patients who received chemotherapy was higher in the low-m^6^Ascore group than in the high-m^6^Ascore group (Du et al., 2021). These results suggest that m^6^A RNA modification has potential clinical implications in cancer patients, indicating their promising implications in improving cancer patient treatment outcomes. Nevertheless, the dual role of m^6^A RNA modification in cancers limited their clinical implications in cancers which needs to be solved in the future. Some of the significant studies examining the potential clinical implications of m^6^A RNA modification in cancers are listed in Table 2.

**TABLE 2:** The potential clinical implications of RNA m^6^A modification in cancers.

Tumor types	Regulators	Expressions	Clinical implications	References
Hepatoblastoma	METTL3	Up	High level of METTL3 is associated with continual recurrence and poor prognosis	Liu et al., 2019; Cui et al., 2020
Bladder cancer Gastric cancer Colorectal cancer	METTL3	Up	Increased expression of METTL3 correlated with poor prognosis	Wang et al., 2020c; Liu et al., 2021b
Colorectal cancer	METTL3	Up	High expression of METTL3 in colorectal cancer is positively correlated with better survival	Deng et al., 2019
Osteosarcoma	METTL3 HNRNPA2B1	Up	High level of METTL3 and HNRNPA2B1 are correlated with poor prognosis	Li et al., 2020b
Osteosarcoma	METTL14	Down	Low level of METTL14 is correlated with poor prognosis	Li et al., 2020b
Breast cancer	METTL14	Down	Low level of METTL14 is negatively correlated with the OS and RFS	Gong et al., 2020
ccRCC	METTL14	Down	High level of METTL14 exhibits as a favorable prognostic factor	Wang Y. et al., 2021
Breast cancer	ZC3H13	Down	Low levels of ZC3H13 is negatively correlated with the OS and PFS	Gong et al., 2020
Acute myeloid leukemia Colon cancer	ALKBH5	Up	Over-expressed ALKBH5 is correlates with poor prognosis	Shen et al., 2020; Huang et al., 2021
HCC Gastric cancer	YTHDF1	Up	Up-regulated YTHDF1 is associated with poor OS	Liu et al., 2020; Pi et al., 2021
Hepatoblastoma HCC	YTHDF2	Up	Over-expressed YTHDF2 is connection with poor prognostic clinical outcomes	Liu et al., 2019; Shao et al., 2020
Osteosarcoma	YTHDF3	Up	High level of YTHDF3 is correlated with poor prognosis	Li et al., 2020b
Head and neck squamous cell carcinoma	YTHDC2	Down	Lower level of YTHDC2 indicates poorer OS and PFS	Li et al., 2020d
Endometrial cancer	YTHDC2	Up	Higher expressions of YTHDC2 is closely associated with worse outcomes and advanced stage	Zhao et al., 2021
Hepatoblastoma	FTO	Up	Up-regulated FTO is correlated with poor clinical outcomes	Liu et al., 2019
Endometrial cancer Lung cancer Rectum adenocarcinoma Pancreatic cancer Osteosarcoma	FTO	Down	Low expression of FTO is correlated with poor prognosis	Zhuang et al., 2019; Li et al., 2020b
Endometrial cancer	HNRNPA2B1 WTAP VIRMA IGF2BP3 HNRNPC	Up	Higher expressions of HNRNPA2B1, WTAP, VIRMA, IGF2BP3, and HNRNPC are closely associated with worse outcomes and advanced stage	Zhao et al., 2021
Gastric cancer HCC	m^6^Ascore	Up	OS for the low m^6^Ascore group was better than the high m^6^Ascore group	He et al., 2021; Shen et al., 2021a
Glioma	m^6^Ascore	Up	OS of low-grade glioma patients who received chemotherapy in the low-m^6^Ascore group is higher than those in the high-m^6^Ascore group	Du et al., 2021

*Abbreviations: ALKBH: ALKB homolog; ccRCC: clear cell renal cell carcinoma; FTO: fat mass and obesity-associated protein; HCC: hepatocellular carcinoma; HNRNP: heterogeneous nuclear ribonucleoprotein; IGF2BP: insulin-like growth factor-2 mRNA-binding protein; m^6^A: N^6^-methyladenosine; METTL: methyltransferase-like; OS: overall survival; PFS: progression-free survival; VIRMA: vir-like m^6^A methyltransferase associated; WTAP: Wilms’ tumor 1-associated protein; YTHDC: YTH domain-containing protein; YTHDF: YTH domain-containing family; ZC3H13: zinc finger CCCH-type containing 13.*

## Targeting m^6^A RNA Modification as Cancer Immunotherapy

The critical role of m^6^A RNA modification in the immune response in the TME and its confirmed clinical implications in cancer make m^6^A RNA modification an attractive immunotherapy in cancer. Studies have shown that lung adenocarcinomas and lung squamous cell carcinomas with lower expression of METTL3, RBM15, ALKBH5, YTHDC1, YTHDF1, YTHDF2, HNRNPC, and VIRMA are significantly more sensitive to immunotherapy and chemotherapy (Xu et al., 2020a,b). Furthermore, studies indicate that reversing the dysregulation of m^6^A RNA modification could promote the effectiveness of immunotherapy in cancer. For example, loss of METTL3 and METTL14 expression increases the response to anti-PD-1 treatment in colorectal cancer with low mutational burden (Wang et al., 2020b). Ablation of METTL3 expression in myeloid cell impairs anti-PD-1 therapeutic efficacy in B16 melanoma (Yin et al., 2021). Deletion of ALKBH5 can sensitize tumors to anti-PD-1 therapy, reduce tumor growth, and prolong mouse survival during GVAX/anti-PD-1 treatment by inhibiting the composition of tumor-infiltrating Tregs and MDSCs *in vitro* and *in vivo*, while melanoma patients harboring ALKBH5 deletion/mutation are more sensitive to anti-PD-1 therapy (Li et al., 2020c). Moreover, the therapeutic effect of anti-PD-L1 is elevated in YTHDF1-deficient mice, suggesting that YTHDF1 is a promising therapeutic target for immunotherapy in combination with checkpoint inhibitors (Han D. et al., 2019). The knockdown of FTO was shown to inhibit the metabolic barrier for CD8^+^ T-cell activation, promoted CD8^+^ T-cell infiltration in tumors, and synergized with anti-PD-L1 treatment (Samstein et al., 2019). In keeping with this, FTO knockdown sensitized melanoma cells to IFN-γ and anti-PD-1 treatment by increasing YTHDF2-dependent PD-1, CXCR4, and SOX10 RNA decay in mice (Yang et al., 2019).

Recently, it was suggested that quantification of the m^6^Ascore could predict the clinical response of cancer patients to immunotherapy. For instance, in colon cancer, Chong et al. (2021) found that cancers with a lower m^6^Ascore show better clinical responses to anti-PD-1, anti-CTLA-4, and anti-PD-L1 therapies. ccRCC patients receiving anti-PD-1, the low m^6^Ascore group presented an apparently prolonged survival (Zhong et al., 2021). Li H. et al. (2021) further validated that a low m^6^Ascore in kidney renal clear cell carcinoma patients indicates an inflammatory phenotype and higher sensitivity to anticancer immunotherapy. Zhou et al. (2021) also confirmed that m^6^Ascore-low pancreatic cancer patients have higher response rates to anti-PD-1 and anti-CTLA-4 treatments. Of note, an RNA modification writer score model was constructed by Chen et al. (2021) recently, which is based on differentially expressed genes responsible for RNA modification patterns and quantifies the RNA modification-related subtypes of individual tumors. The authors found that colorectal cancer patients with a low writer score in an anti-PD-L1 cohort presented significant clinical benefits and had a dramatically prolonged OS (Chen et al., 2021). Notably, we found that in some m^6^A regulators, such as YTHDF1 and YTHDF2, their lower expressions are both more sensitive to immunotherapy, suggesting a possible cooperative role in tumor immunotherapy, which needs to be explored in future studies. Some of the most important studies examining m^6^A RNA modification as a potential target for cancer immunotherapy are listed in Table 3.

**TABLE 3:** Targeting RNA m^6^A modification as cancer immunotherapy.

Tumor types	Regulators	Immunotherapy	References
Lung adenocarcinoma	METTL3	Lower expressions of METTL3 is more sensitive to immunotherapy	Xu et al., 2020a
Low mutation burden of colorectal cancer	METTL3 METTL14	Loss of METTL3 and METTL14 increase response to anti-PD-1 treatment	Wang et al., 2020b
Melanoma	METTL3	Ablation of METTL3 in myeloid cells impairs anti-PD-1 therapeutic efficacy	Yin et al., 2021
Melanoma Non-small cell lung cancer	FTO	Knockdown of FTO synergizes with anti-PD-L1 treatment	Samstein et al., 2019
Melanoma	FTO	Knockdown of FTO sensitizes melanoma to anti-PD-1	Yang et al., 2019
Melanoma	ALKBH5	Melanoma patients harboring ALKBH5 deletion/mutation are correlated with more sensitive to anti-PD-1 therapy	Li et al., 2020c
Lung adenocarcinoma	ALKBH5 RBM15 YTHDC1 YTHDF1 YTHDF2	Lower expressions of ALKBH5, RBM15, YTHDC1, YTHDF1, and YTHDF2 are more sensitive to immunotherapy	Xu et al., 2020a
Melanoma Colon cancer	YTHDF1	The therapeutic effect of anti-PD-L1 is elevated in YTHDF1 deficient mice	Han D. et al., 2019
Lung squamous cell carcinoma	HNRNPC	Lower expressions of HNRNPC and VIRMA are more sensitive to immunotherapy and chemotherapy	Xu et al., 2020b
Colon cancer	m^6^Ascore	Lower m^6^Ascore showed a better clinical benefits to anti-PD-1, anti-CTLA-4, and anti-PD-L1 therapies	Chong et al., 2021
ccRCC	m^6^Ascore	Low m^6^Ascore group presents a apparently prolonged survival in the anti-PD-1ccRCC patient	Zhong et al., 2021
Kidney renal clear cell carcinoma	m^6^Ascore	Low m^6^Ascore indicates an inflammatory phenotype and more sensitive to anticancer immunotherapy	Li H. et al., 2021
Pancreatic cancer	m^6^Ascore	m^6^Ascore-low pancreatic cancer patients have higher response rates to anti-PD-1and anti-CTLA-4 treatments	Zhou et al., 2021
Colorectal cancer	“Writer” score	Low “writer” score present significant clinical benefits and have a dramatically prolonged OS in anti-PD-L1 cohort	Chen et al., 2021

*Abbreviations: ALKBH: ALKB homolog; ccRCC: clear cell renal cell carcinoma; CTLA-4: cytotoxic T cell lymphocyte-associated protein 4; FTO: fat mass and obesity-associated protein; HNRNP: heterogeneous nuclear ribonucleoprotein; m^6^A: N^6^-methyladenosine; METTL: methyltransferase-like; OS: overall survival; PD-1: programmed death receptor 1; PD-L1: programmed death receptor ligand 1; RBM: RNA-binding motif; VIRMA: vir-like m6A methyltransferase associated; YTHDC: YTH domain-containing protein; YTHDF: YTH domain-containing family.*

## Conclusion and Future Perspectives

Within the past decade, m^6^A RNA modification has been identified as a novel emerging layer of posttranscriptional regulation controlling gene expression in eukaryotes. Currently, it is clear that m^6^A RNA modification exhibits essential roles in almost all bioprocesses, including the immune response in cancers. In our present review, we have focused on discussing the underlying mechanisms whereby m^6^A RNA modification is implicated in cold tumor formation. We have also discussed the potential clinical implications and immunotherapeutic strategies of targeting m^6^A RNA modification in cancer. Indeed, m^6^A RNA modification is involved in cold tumor formation by regulating the immune cells in the TME, targeting tumor-cell-intrinsic pathways, facilitating the action of soluble inhibitory mediators in the TME, increasing metabolic competition in the TME, and affecting the tumor mutational burden. Furthermore, many m^6^A RNA modification regulators (m^6^A writers, erasers, and readers) have potential clinical applications as diagnostic and prognostic biomarkers for different types of cancer. In addition, targeting m^6^A RNA modification regulators could sensitize cancers to immunotherapy. Thus, targeting m^6^A RNA modification is a promising immunotherapeutic approach for turning cold tumors into hot ones.

Although tremendous progress has been achieved on understanding m^6^A RNA modification and their role in diseases, a complete understanding of the mechanisms is far away, and especially, their implications in cancers is our concern. The present researches show that the abnormal level of m^6^A regulators are intimately associated with the prognosis of tumors, indicating their promising implications in improving cancer patient treatment outcomes, although it has been demonstrated that targeting RNA m^6^A modification could be the optional combination therapy in cancer immunotherapy, the limitation is that except for the role of RNA m^6^A modification in immune response, their functions in tumor development should be taken into consideration, which could be a cause of immunotherapeutic resistance or insensitivity. For example, PD-1/PD-L1 acts as a tumor suppressor and mediates resistance to PD-1 blockade therapy in tumor (Wang et al., 2020e). Therefore, we believe that future research on m^6^A RNA modification should focus on several aspects. First, some specific m^6^A RNA modification regulators play opposite roles in different cancers, indicating that the exact role of m^6^A RNA modification regulators is cell or tissue dependent (Deng et al., 2018; Zeng et al., 2020). Consequently, defining the context-specific role of m^6^A RNA modification regulators in cancers and their mechanisms will be crucial to direct specific m^6^A RNA modification regulator-based therapeutic interventions in the future. Second, we know that m^6^A RNA modification is found not only in mRNAs but also in non-coding RNAs, and that non-coding RNAs play critical roles in the immune response and immunotherapy in cancers (Atianand et al., 2017; Huang et al., 2020a); therefore, future studies focused on m^6^A-related non-coding RNAs in cancer will contribute toward the development of more effective and novel cancer immunotherapies (Chen et al., 2020; Xu et al., 2021a). Third, studies evaluating the use of m^6^A RNA modification as cancer immunotherapy have mainly focused on regulating m^6^A RNA modification regulators through transfection experiments, which are difficult to translate to clinical trials or clinical practice; therefore, m^6^A RNA modification regulator agonists or antagonists should be searched in the future (Su et al., 2018). Lastly, considering the toxic side effects of cancer immunotherapy, target carrier material should be developed to carry immunotherapeutics including m^6^A modification RNA regulators that augment antitumor immune responses with reduced toxicity and side effects (Zeng et al., 2021).

## Author Contributions

LZ and JuZ collected the data, finished the manuscript, and prepared the figures and tables. YC and BW gave constructive guidance. SZ, XL, JiZ, WW, YF, and SS participated in the design of this review. All authors read and approved the final manuscript.

## Conflict of Interest

The authors declare that the research was conducted in the absence of any commercial or financial relationships that could be construed as a potential conflict of interest.

## Publisher’s Note

All claims expressed in this article are solely those of the authors and do not necessarily represent those of their affiliated organizations, or those of the publisher, the editors and the reviewers. Any product that may be evaluated in this article, or claim that may be made by its manufacturer, is not guaranteed or endorsed by the publisher.

## Abbreviations

3′ -UTR: 3′ -untranslated region
α KG: α-ketoglutarate
A: adenosine
ALDOA: abnormal expression of aldolase A
ALKBH5: ALKB homolog 5
C: cytidine
CCR4-NOT: carbon catabolite repressor 4-negative on TATA
circRNAs: circular RNAs
CSF-1R: colony stimulating factor-1 receptor
CTLA-4: cytotoxic T-cell lymphocyte-associated protein 4
DCs: dendritic cells
ECM: extracellular matrix
eIF: eukaryotic translation initiation factor
EMT: epithelial-mesenchymal transition
f^6^A: N^6^-formyladenosine
FDA: Food and Drug Administration
FTO: fat mass and obesity-associated protein
G: guanosine
H3K36me3: histone H3 lysine 36 trimethylation
HCC: hepatocellular carcinoma
HDGF: hepatoma-derived growth factor
HER2: human epidermal growth factor receptor 2
HIF: hypoxia-inducible factor
hm^6^A: N^6^-hydroxymethyladenosine
HNRNP: heterogeneous nuclear ribonucleoprotein
IDO1: indoleamine-pyrrole 2,3-dioxygenase 1
IFN- γ: interferon γ
IGF2BP: insulin-like growth factor-2 mRNA-binding protein
IL: interleukin
IRAK3: IL-1 receptor-associated kinase 3
LAG-3: lymphocyte activation gene-3
lncRNAs: long non-coding RNAs
m^1^A: N^1^-methyladenosine
m^6^A: N^6^-methyladenosine
m^6^A_m_: N^6^ 2′ -O-dimethyladenosine
MDSCs: myeloid-derived suppressor cells
MEIOC: meiosis-specific coiled-coil domain
METTL: methyltransferase-like
mRNA: messenger RNA
MHC I: major histocompatibility complex class I
mRNP: messenger ribonucleoprotein
MTC: methyltransferase complex
NF- κ B: nuclear factor kappa-B
NGS: next-generation sequencing
OS: overall survival
PABP: poly(A) binding protein
PD-1: programmed death receptor 1
PD-L1: programmed death receptor ligand 1
RBM 4: RNA-binding motif 4
PFS: progression-free survival
ROC: receiver operating characteristic curve
rRNAs: ribosomal RNAs
SAH: *S*-adenosyl homocysteine
SAM: *S*-adenosylmethionine
SASP: senescence-associated secretory phenotype
snRNAs: small nuclear RNAs
snoRNAs: small nucleolar RNAs
SRSF: splicing factor serine- and arginine-rich splicing factor
TAMs: tumor-associated macrophages
TEK: tyrosine kinase endothelial
TGF- β: transforming growth factor- β
TIGIT: T-cell immunoglobulin and ITIM domain
TILs: tumor-infiltrating lymphocytes
TIM-3: T-cell immunoglobulin and mucin-domain containing-3
TLR4: toll-like receptors 4
TME: tumor microenvironment
Tregs: regulatory T cells
U: uridine
VEGF: vascular endothelial growth factor
VHL: von Hippel-Lindau
VIRMA: vir-like m^6^A methyltransferase associated
VISTA: V-domain Ig suppressor of T cell activation
WTAP: Wilms’ tumor 1-associated protein
YTH: YT521-B homology
YTHDC2: YTH domain-containing protein 2
YTHDFs: YTH domain-containing family
ZC3H13: zinc finger CCCH-type containing 13
ZCCHC4: zinc finger CCHC-type containing 4.

## References

[B1] Aggen D. H. Drake C. G. Rini B. I. (2020). Targeting PD-1 or PD-L1 in metastatic kidney cancer: combination therapy in the first-line setting. *Clin. Cancer Res.* 26 2087–2095. 10.1158/1078-0432.CCR-19-3323 31948999

[B2] Alarcon C. R. Goodarzi H. Lee H. Liu X. Tavazoie S. Tavazoie S. F. (2015). HNRNPA2B1 is a mediator of m(6)A-Dependent nuclear RNA processing events. *Cell* 162 1299–1308. 10.1016/j.cell.2015.08.011 26321680 PMC4673968

[B3] Atianand M. K. Caffrey D. R. Fitzgerald K. A. (2017). Immunobiology of long noncoding RNAs. *Annu. Rev. Immunol.* 35 177–198. 10.1146/annurev-immunol-041015-055459 28125358 PMC6449690

[B4] Bai Y. Yang C. Wu R. Huang L. Song S. Li W. (2019). YTHDF1 regulates tumorigenicity and cancer stem cell-Like activity in human colorectal carcinoma. *Front. Oncol.* 9:332. 10.3389/fonc.2019.00332 31131257 PMC6509179

[B5] Balkwill F. Mantovani A. (2001). Inflammation and cancer: back to Virchow? *Lancet* 357 539–545. 10.1016/S0140-6736(00)04046-0 11229684

[B6] Binnewies M. Roberts E. W. Kersten K. Chan V. Fearon D. F. Merad M. (2018). Understanding the tumor immune microenvironment (TIME) for effective therapy. *Nat. Med.* 24 541–550. 10.1038/s41591-018-0014-x 29686425 PMC5998822

[B7] Bodi Z. Fray R. G. (2017). Detection and quantification of n (6)-methyladenosine in messenger RNA by TLC. *Methods Mol. Biol.* 1562 79–87. 10.1007/978-1-4939-6807-7_6 28349455

[B8] Bokar J. A. Shambaugh M. E. Polayes D. Matera A. G. Rottman F. M. (1997). Purification and cDNA cloning of the AdoMet-binding subunit of the human mRNA (N6-adenosine)-methyltransferase. *RNA* 3 1233–1247. 9409616 PMC1369564

[B9] Camus M. Tosolini M. Mlecnik B. Pages F. Kirilovsky A. Berger A. (2009). Coordination of intratumoral immune reaction and human colorectal cancer recurrence. *Cancer Res.* 69 2685–2693. 10.1158/0008-5472.CAN-08-2654 19258510

[B10] Cannarile M. A. Weisser M. Jacob W. Jegg A. M. Ries C. H. Ruttinger D. (2017). Colony-stimulating factor 1 receptor (CSF1R) inhibitors in cancer therapy. *J. Immunother. Cancer* 5:53. 10.1186/s40425-017-0257-y 28716061 PMC5514481

[B11] Cebrian I. Visentin G. Blanchard N. Jouve M. Bobard A. Moita C. (2011). Sec22b regulates phagosomal maturation and antigen crosspresentation by dendritic cells. *Cell* 147 1355–1368. 10.1016/j.cell.2011.11.021 22153078

[B12] Chen H. Yao J. Bao R. Dong Y. Zhang T. Du Y. (2021). Cross-talk of four types of RNA modification writers defines tumor microenvironment and pharmacogenomic landscape in colorectal cancer. *Mol. Cancer* 20:29. 10.1186/s12943-021-01322-w 33557837 PMC7869236

[B13] Chen K. Wei Z. Zhang Q. Wu X. Rong R. Lu Z. (2019). WHISTLE: a high-accuracy map of the human N6-methyladenosine (m6A) epitranscriptome predicted using a machine learning approach. *Nucleic Acids Res.* 47:e41. 10.1093/nar/gkz074 30993345 PMC6468314

[B14] Chen W. Ten Dijke P. (2016). Immunoregulation by members of the TGFbeta superfamily. *Nat. Rev. Immunol.* 16 723–740. 10.1038/nri.2016.112 27885276

[B15] Chen Y. Lin Y. Shu Y. He J. Gao W. (2020). Interaction between N(6)-methyladenosine (m(6)A) modification and noncoding RNAs in cancer. *Mol. Cancer* 19:94. 10.1186/s12943-020-01207-4 32443966 PMC7243333

[B16] Cheon H. Borden E. C. Stark G. R. (2014). Interferons and their stimulated genes in the tumor microenvironment. *Semin. Oncol.* 41 156–173. 10.1053/j.seminoncol.2014.02.002 24787290 PMC4118773

[B17] Chong W. Shang L. Liu J. Fang Z. Du F. Wu H. (2021). M(6)A regulator-based methylation modification patterns characterized by distinct tumor microenvironment immune profiles in colon cancer. *Theranostics* 11 2201–2217. 10.7150/thno.52717 33500720 PMC7797678

[B18] Cui X. Wang Z. Li J. Zhu J. Ren Z. Zhang D. (2020). Cross talk between RNA N6-methyladenosine methyltransferase-like 3 and miR-186 regulates hepatoblastoma progression through Wnt/beta-catenin signalling pathway. *Cell Prolif.* 53:e12768. 10.1111/cpr.12768 31967701 PMC7106953

[B19] Dawson M. A. (2017). The cancer epigenome: concepts, challenges, and therapeutic opportunities. *Science* 355 1147–1152. 10.1126/science.aam7304 28302822

[B20] Deng R. Cheng Y. Ye S. Zhang J. Huang R. Li P. (2019). M(6)A methyltransferase METTL3 suppresses colorectal cancer proliferation and migration through p38/ERK pathways. *Onco Targets Ther.* 12 4391–4402. 10.2147/OTT.S201052 31239708 PMC6556107

[B21] Deng X. Su R. Weng H. Huang H. Li Z. Chen J. (2018). RNA N(6)-methyladenosine modification in cancers: current status and perspectives. *Cell Res.* 28 507–517. 10.1038/s41422-018-0034-6 29686311 PMC5951805

[B22] Desrosiers R. Friderici K. Rottman F. (1974). Identification of methylated nucleosides in messenger RNA from Novikoff hepatoma cells. *Proc. Natl. Acad. Sci. U.S.A.* 71 3971–3975. 10.1073/pnas.71.10.3971 4372599 PMC434308

[B23] Dominissini D. Moshitch-Moshkovitz S. Schwartz S. Salmon-Divon M. Ungar L. Osenberg S. (2012). Topology of the human and mouse m6A RNA methylomes revealed by m6A-seq. *Nature* 485 201–206. 10.1038/nature11112 22575960

[B24] Du H. Zhao Y. He J. Zhang Y. Xi H. Liu M. (2016). YTHDF2 destabilizes m(6)A-containing RNA through direct recruitment of the CCR4-NOT deadenylase complex. *Nat. Commun.* 7:12626. 10.1038/ncomms12626 27558897 PMC5007331

[B25] Du J. Ji H. Ma S. Jin J. Mi S. Hou K. (2021). M6A regulator-mediated methylation modification patterns and characteristics of immunity and stemness in low-grade glioma. *Brief. Bioinform.* bbab013. 10.1093/bib/bbab013 33594424

[B26] Duan Q. Zhang H. Zheng J. Zhang L. (2020). Turning cold into hot: firing up the tumor microenvironment. *Trends Cancer* 6 605–618. 10.1016/j.trecan.2020.02.022 32610070

[B27] Fridman W. H. Pages F. Sautes-Fridman C. Galon J. (2012). The immune contexture in human tumours: impact on clinical outcome. *Nat. Rev. Cancer* 12 298–306. 10.1038/nrc3245 22419253

[B28] Fukumoto T. Zhu H. Nacarelli T. Karakashev S. Fatkhutdinov N. Wu S. (2019). N(6)-Methylation of adenosine of FZD10 mRNA contributes to PARP inhibitor resistance. *Cancer Res.* 79 2812–2820. 10.1158/0008-5472.CAN-18-3592 30967398 PMC6548690

[B29] Gajewski T. F. (2015). The next hurdle in cancer immunotherapy: overcoming the non-T-cell-inflamed tumor microenvironment. *Semin. Oncol.* 42 663–671. 10.1053/j.seminoncol.2015.05.011 26320069 PMC4555998

[B30] Gajewski T. F. Corrales L. Williams J. Horton B. Sivan A. Spranger S. (2017). Cancer immunotherapy targets based on understanding the T cell-inflamed versus non-T cell-inflamed tumor microenvironment. *Adv. Exp. Med. Biol.* 1036 19–31. 10.1007/978-3-319-67577-0_2 29275462 PMC6693322

[B31] Galon J. Bruni D. (2019). Approaches to treat immune hot, altered and cold tumours with combination immunotherapies. *Nat. Rev. Drug Discov.* 18 197–218. 10.1038/s41573-018-0007-y 30610226

[B32] Galon J. Fridman W. H. Pages F. (2007). The adaptive immunologic microenvironment in colorectal cancer: a novel perspective. *Cancer Res.* 67 1883–1886. 10.1158/0008-5472.CAN-06-4806 17332313

[B33] Garcia-Campos M. A. Edelheit S. Toth U. Safra M. Shachar R. Viukov S. (2019). Deciphering the “m(6)A Code” via antibody-independent quantitative profiling. *Cell* 178 731–747. 10.1016/j.cell.2019.06.013 31257032

[B34] Gong P. J. Shao Y. C. Yang Y. Song W. J. He X. Zeng Y. F. (2020). Analysis of N6-methyladenosine methyltransferase reveals METTL14 and ZC3H13 as tumor suppressor genes in breast cancer. *Front. Oncol.* 10:578963. 10.3389/fonc.2020.578963 33363011 PMC7757663

[B35] Gulati P. Avezov E. Ma M. Antrobus R. Lehner P. O’Rahilly S. (2014). Fat mass and obesity-related (FTO) shuttles between the nucleus and cytoplasm. *Biosci. Rep.* 34:e00144. 10.1042/BSR20140111 25242086 PMC4206862

[B36] Han B. Yan S. Wei S. Xiang J. Liu K. Chen Z. (2020). YTHDF1-mediated translation amplifies Wnt-driven intestinal stemness. *EMBO Rep.* 21:e49229. 10.15252/embr.201949229 32064749 PMC7132202

[B37] Han D. Liu J. Chen C. Dong L. Liu Y. Chang R. (2019). Anti-tumour immunity controlled through mRNA m(6)A methylation and YTHDF1 in dendritic cells. *Nature* 566 270–274. 10.1038/s41586-019-0916-x 30728504 PMC6522227

[B38] Han J. Wang J. Z. Yang X. Yu H. Zhou R. Lu H. C. (2019). METTL3 promote tumor proliferation of bladder cancer by accelerating pri-miR221/222 maturation in m6A-dependent manner. *Mol. Cancer* 18:110. 10.1186/s12943-019-1036-9 31228940 PMC6588935

[B39] He X. Tan L. Ni J. Shen G. (2021). Expression pattern of m(6)A regulators is significantly correlated with malignancy and antitumor immune response of breast cancer. *Cancer Gene Ther.* 28 188–196. 10.1038/s41417-020-00208-1 32759989 PMC8057950

[B40] Hegde P. S. Karanikas V. Evers S. (2016). The where, the when, and the how of immune monitoring for cancer immunotherapies in the era of checkpoint inhibition. *Clin. Cancer Res.* 22 1865–1874. 10.1158/1078-0432.CCR-15-1507 27084740

[B41] Hou H. Zhao H. Yu X. Cong P. Zhou Y. Jiang Y. (2020). METTL3 promotes the proliferation and invasion of esophageal cancer cells partly through AKT signaling pathway. *Pathol. Res. Pract.* 216:153087. 10.1016/j.prp.2020.153087 32825955

[B42] Hsu P. J. Zhu Y. Ma H. Guo Y. Shi X. Liu Y. (2017). Ythdc2 is an N(6)-methyladenosine binding protein that regulates mammalian spermatogenesis. *Cell Res.* 27 1115–1127. 10.1038/cr.2017.99 28809393 PMC5587856

[B43] Huang A. C. Postow M. A. Orlowski R. J. Mick R. Bengsch B. Manne S. (2017). T-cell invigoration to tumour burden ratio associated with anti-PD-1 response. *Nature* 545 60–65. 10.1038/nature22079 28397821 PMC5554367

[B44] Huang H. Weng H. Chen J. (2020a). M(6)A modification in coding and non-coding RNAs: Roles and therapeutic implications in cancer. *Cancer Cell* 37, 270–288. 10.1016/j.ccell.2020.02.004 32183948 PMC7141420

[B45] Huang H. Weng H. Chen J. (2020b). The biogenesis and precise control of RNA m(6)A methylation. *Trends Genet.* 36 44–52. 10.1016/j.tig.2019.10.011 31810533 PMC6925345

[B46] Huang H. Weng H. Zhou K. Wu T. Zhao B. S. Sun M. (2019). Histone H3 trimethylation at lysine 36 guides m(6)A RNA modification co-transcriptionally. *Nature* 567 414–419. 10.1038/s41586-019-1016-7 30867593 PMC6438714

[B47] Huang L. Zhu J. Kong W. Li P. Zhu S. (2021). Expression and prognostic characteristics of m6A RNA methylation regulators in colon cancer. *Int. J. Mol. Sci.* 22:2134. 10.3390/ijms22042134 33670062 PMC7926939

[B48] Huangfu N. Zheng W. Xu Z. Wang S. Wang Y. Cheng J. (2020). RBM4 regulates M1 macrophages polarization through targeting STAT1-mediated glycolysis. *Int. Immunopharmacol.* 83:106432. 10.1016/j.intimp.2020.106432 32248017

[B49] Jia G. Fu Y. Zhao X. Dai Q. Zheng G. Yang Y. (2011). N6-methyladenosine in nuclear RNA is a major substrate of the obesity-associated FTO. *Nat. Chem. Biol.* 7 885–887. 10.1038/nchembio.687 22002720 PMC3218240

[B50] Jung G. Hernandez-Illan E. Moreira L. Balaguer F. Goel A. (2020). Epigenetics of colorectal cancer: biomarker and therapeutic potential. *Nat. Rev. Gastroenterol. Hepatol.* 17 111–130. 10.1038/s41575-019-0230-y 31900466 PMC7228650

[B51] Kataoka K. (2019). [Genetic alterations involving PD-L1/PD-L2 in human malignancies]. *Gan Kagaku Ryoho* 46 841–844. 31189800

[B52] Kedia-Mehta N. Finlay D. K. (2019). Competition for nutrients and its role in controlling immune responses. *Nat. Commun.* 10:2123. 10.1038/s41467-019-10015-4 31073180 PMC6509329

[B53] Kim G. W. Imam H. Khan M. Mir S. A. Kim S. J. Yoon S. K. (2021). HBV-induced increased N6 methyladenosine modification of PTEN RNA affects innate immunity and contributes to HCC. *Hepatology* 73 533–547. 10.1002/hep.31313 32394474 PMC7655655

[B54] Knuckles P. Lence T. Haussmann I. U. Jacob D. Kreim N. Carl S. H. (2018). Zc3h13/Flacc is required for adenosine methylation by bridging the mRNA-binding factor Rbm15/Spenito to the m(6)A machinery component Wtap/Fl(2)d. *Genes Dev.* 32 415–429. 10.1101/gad.309146.117 29535189 PMC5900714

[B55] Leismann J. Spagnuolo M. Pradhan M. Wacheul L. Vu M. A. Musheev M. (2020). The 18S ribosomal RNA m(6) a methyltransferase Mettl5 is required for normal walking behavior in *Drosophila*. *EMBO Rep.* 21:e49443. 10.15252/embr.201949443 32350990 PMC7332798

[B56] Li A. Chen Y. S. Ping X. L. Yang X. Xiao W. Yang Y. (2017). Cytoplasmic m(6)A reader YTHDF3 promotes mRNA translation. *Cell Res.* 27 444–447. 10.1038/cr.2017.10 28106076 PMC5339832

[B57] Li H. Hu J. Yu A. Othmane B. Guo T. Liu J. (2021). RNA modification of N6-Methyladenosine predicts immune phenotypes and therapeutic opportunities in kidney renal clear cell carcinoma. *Front. Oncol.* 11:642159. 10.3389/fonc.2021.642159 33816290 PMC8013979

[B58] Li H. B. Tong J. Zhu S. Batista P. J. Duffy E. E. Zhao J. (2017). M(6)A mRNA methylation controls T cell homeostasis by targeting the IL-7/STAT5/SOCS pathways. *Nature* 548 338–342. 10.1038/nature23450 28792938 PMC5729908

[B59] Li J. Chen F. Peng Y. Lv Z. Lin X. Chen Z. (2020a). N6-methyladenosine regulates the expression and secretion of TGFbeta1 to affect the epithelial-mesenchymal transition of cancer cells. *Cells* 9:296. 10.3390/cells9020296 31991845 PMC7072279

[B60] Li J. Rao B. Yang J. Liu L. Huang M. Liu X. (2020b). Dysregulated m6A-related regulators are associated with tumor metastasis and poor prognosis in osteosarcoma. *Front. Oncol.* 10:769. 10.3389/fonc.2020.00769 32582536 PMC7280491

[B61] Li N. Kang Y. Wang L. Huff S. Tang R. Hui H. (2020c). ALKBH5 regulates anti-PD-1 therapy response by modulating lactate and suppressive immune cell accumulation in tumor microenvironment. *Proc. Natl. Acad. Sci. U.S.A.* 117 20159–20170. 10.1073/pnas.1918986117 32747553 PMC7443867

[B62] Li X. Luo L. Jiang M. Zhu C. Shi Y. Zhang J. (2021). Cocktail strategy for ‘cold’ tumors therapy via active recruitment of CD8+ T cells and enhancing their function. *J. Control. Release* 334 413–426. 10.1016/j.jconrel.2021.05.002 33964366

[B63] Li Y. Zheng J. N. Wang E. H. Gong C. J. Lan K. F. Ding X. (2020d). The m6A reader protein YTHDC2 is a potential biomarker and associated with immune infiltration in head and neck squamous cell carcinoma. *PeerJ* 8:e10385. 10.7717/peerj.10385 33304653 PMC7700739

[B64] Ling C. Ronn T. (2019). Epigenetics in human obesity and type 2 diabetes. *Cell Metab.* 29 1028–1044. 10.1016/j.cmet.2019.03.009 30982733 PMC6509280

[B65] Liu J. Eckert M. A. Harada B. T. Liu S. M. Lu Z. Yu K. (2018). M(6)A mRNA methylation regulates AKT activity to promote the proliferation and tumorigenicity of endometrial cancer. *Nat. Cell Biol.* 20 1074–1083. 10.1038/s41556-018-0174-4 30154548 PMC6245953

[B66] Liu L. Wang J. Sun G. Wu Q. Ma J. Zhang X. (2019). M(6)A mRNA methylation regulates CTNNB1 to promote the proliferation of hepatoblastoma. *Mol. Cancer* 18:188. 10.1186/s12943-019-1119-7 31870368 PMC6927193

[B67] Liu N. Dai Q. Zheng G. He C. Parisien M. Pan T. (2015). N(6)-methyladenosine-dependent RNA structural switches regulate RNA-protein interactions. *Nature* 518 560–564. 10.1038/nature14234 25719671 PMC4355918

[B68] Liu P. Li F. Lin J. Fukumoto T. Nacarelli T. Hao X. (2021a). M(6)A-independent genome-wide METTL3 and METTL14 redistribution drives the senescence-associated secretory phenotype. *Nat. Cell Biol.* 23 355–365. 10.1038/s41556-021-00656-3 33795874 PMC8035315

[B69] Liu X. Qin J. Gao T. Li C. He B. Pan B. (2020). YTHDF1 facilitates the progression of hepatocellular carcinoma by promoting FZD5 mRNA translation in an m6A-dependent manner. *Mol. Ther. Nucleic Acids* 22 750–765. 10.1016/j.omtn.2020.09.036 33230473 PMC7595883

[B70] Liu X. Su K. Sun X. Jiang Y. Wang L. Hu C. (2021b). Sec62 promotes stemness and chemoresistance of human colorectal cancer through activating Wnt/beta-catenin pathway. *J. Exp. Clin. Cancer Res.* 40:132. 10.1186/s13046-021-01934-6 33858476 PMC8051072

[B71] Liu X. Wang P. Teng X. Zhang Z. Song S. (2021c). Comprehensive analysis of expression regulation for RNA m6A regulators with clinical significance in human cancers. *Front. Oncol.* 11:624395. 10.3389/fonc.2021.624395 33718187 PMC7946859

[B72] Liu Y. Liang G. Xu H. Dong W. Dong Z. Qiu Z. (2021d). Tumors exploit FTO-mediated regulation of glycolytic metabolism to evade immune surveillance. *Cell Metab.* 33 1221–1233. 10.1016/j.cmet.2021.04.001 33910046

[B73] Liu Y. Wang R. Zhang L. Li J. Lou K. Shi B. (2017). The lipid metabolism gene FTO influences breast cancer cell energy metabolism via the PI3K/AKT signaling pathway. *Oncol. Lett.* 13 4685–4690. 10.3892/ol.2017.6038 28599470 PMC5452952

[B74] Lohmueller J. Finn O. J. (2017). Current modalities in cancer immunotherapy: immunomodulatory antibodies, CARs and vaccines. *Pharmacol. Ther.* 178 31–47. 10.1016/j.pharmthera.2017.03.008 28322974 PMC5600680

[B75] Ma H. Wang X. Cai J. Dai Q. Natchiar S. K. Lv R. (2019). N(6-)Methyladenosine methyltransferase ZCCHC4 mediates ribosomal RNA methylation. *Nat. Chem. Biol.* 15 88–94. 10.1038/s41589-018-0184-3 30531910 PMC6463480

[B76] MacDonald B. T. He X. (2012). Frizzled and LRP5/6 receptors for Wnt/beta-catenin signaling. *Cold Spring Harb. Perspect. Biol.* 4:a007880. 10.1101/cshperspect.a007880 23209147 PMC3504444

[B77] Meyer K. D. Saletore Y. Zumbo P. Elemento O. Mason C. E. Jaffrey S. R. (2012). Comprehensive analysis of mRNA methylation reveals enrichment in 3′ UTRs and near stop codons. *Cell* 149 1635–1646. 10.1016/j.cell.2012.05.003 22608085 PMC3383396

[B78] Mo P. Xie S. Cai W. Ruan J. Du Q. Ye J. (2020). N(6)-methyladenosine (m(6)A) RNA methylation signature as a predictor of stomach adenocarcinoma outcomes and its association with immune checkpoint molecules. *J. Int. Med. Res.* 48:1220750957. 10.1177/0300060520951405 32972288 PMC7522833

[B79] Mok S. Koya R. C. Tsui C. Xu J. Y. Robert L. Wu L. (2014). Inhibition of CSF-1 receptor improves the antitumor efficacy of adoptive cell transfer immunotherapy. *Cancer Res.* 74 153–161. 10.1158/0008-5472.CAN-13-1816 24247719 PMC3947337

[B80] Niu Y. Lin Z. Wan A. Sun L. Yan S. Liang H. (2021). Loss-of-function genetic screening identifies ALDOA as an essential driver for liver cancer cell growth under hypoxia. *Hepatology.* 10.1002/hep.31846 33813748 PMC8518375

[B81] Ochoa D. O. M. Navarro R. B. Zimmermann S. Coukos G. (2020). Turning up the heat on non-immunoreactive tumours: opportunities for clinical development. *Lancet Oncol.* 21 e419–e430. 10.1016/S1470-2045(20)30234-5 32888471

[B82] Peng W. Li J. Chen R. Gu Q. Yang P. Qian W. (2019). Upregulated METTL3 promotes metastasis of colorectal cancer via miR-1246/SPRED2/MAPK signaling pathway. *J. Exp. Clin. Cancer Res.* 38:393. 10.1186/s13046-019-1408-4 31492150 PMC6729001

[B83] Pi J. Wang W. Ji M. Wang X. Wei X. Jin J. (2021). YTHDF1 promotes gastric carcinogenesis by controlling translation of FZD7. *Cancer Res.* 81 2651–2665. 10.1158/0008-5472.CAN-20-0066 32788173

[B84] Ping X. L. Sun B. F. Wang L. Xiao W. Yang X. Wang W. J. (2014). Mammalian WTAP is a regulatory subunit of the RNA N6-methyladenosine methyltransferase. *Cell Res.* 24 177–189. 10.1038/cr.2014.3 24407421 PMC3915904

[B85] Qin S. Xu L. Yi M. Yu S. Wu K. Luo S. (2019). Novel immune checkpoint targets: moving beyond PD-1 and CTLA-4. *Mol. Cancer* 18:155. 10.1186/s12943-019-1091-2 31690319 PMC6833286

[B86] Razak A. R. Cleary J. M. Moreno V. Boyer M. Aller E. C. Edenfield W. (2020). Safety and efficacy of AMG 820, an anti-colony-stimulating factor 1 receptor antibody, in combination with pembrolizumab in adults with advanced solid tumors. *J. Immunother. Cancer* 8:e001006. 10.1136/jitc-2020-001006 33046621 PMC7552843

[B87] Roignant J. Y. Soller M. (2017). M(6)A in mRNA: an ancient mechanism for fine-tuning gene expression. *Trends Genet.* 33 380–390. 10.1016/j.tig.2017.04.003 28499622

[B88] Rosenberg S. A. Restifo N. P. (2015). Adoptive cell transfer as personalized immunotherapy for human cancer. *Science* 348 62–68. 10.1126/science.aaa4967 25838374 PMC6295668

[B89] Rotte A. (2019). Combination of CTLA-4 and PD-1 blockers for treatment of cancer. *J. Exp. Clin. Cancer Res.* 38:255. 10.1186/s13046-019-1259-z 31196207 PMC6567914

[B90] Roundtree I. A. Luo G. Z. Zhang Z. Wang X. Zhou T. Cui Y. (2017). YTHDC1 mediates nuclear export of N(6)-methyladenosine methylated mRNAs. *eLife* 6:e31311. 10.7554/eLife.31311 28984244 PMC5648532

[B91] Saletore Y. Meyer K. Korlach J. Vilfan I. D. Jaffrey S. Mason C. E. (2012). The birth of the epitranscriptome: deciphering the function of RNA modifications. *Genome Biol.* 13:175. 10.1186/gb-2012-13-10-175 23113984 PMC3491402

[B92] Samstein R. M. Lee C. H. Shoushtari A. N. Hellmann M. D. Shen R. Janjigian Y. Y. (2019). Tumor mutational load predicts survival after immunotherapy across multiple cancer types. *Nat. Genet.* 51 202–206. 10.1038/s41588-018-0312-8 30643254 PMC6365097

[B93] Shao X. Y. Dong J. Zhang H. Wu Y. S. Zheng L. (2020). Systematic analyses of the role of the reader protein of n (6)-methyladenosine RNA methylation, YTH domain family 2, in liver hepatocellular carcinoma. *Front. Mol. Biosci.* 7:577460. 10.3389/fmolb.2020.577460 33344502 PMC7738478

[B94] Sharma P. Allison J. P. (2015). The future of immune checkpoint therapy. *Science* 348 56–61. 10.1126/science.aaa8172 25838373

[B95] Shen C. Sheng Y. Zhu A. C. Robinson S. Jiang X. Dong L. (2020). RNA demethylase ALKBH5 selectively promotes tumorigenesis and cancer stem cell self-renewal in acute myeloid leukemia. *Cell Stem Cell* 27 64–80.e9. 10.1016/j.stem.2020.04.009 32402250 PMC7335338

[B96] Shen S. Yan J. Zhang Y. Dong Z. Xing J. He Y. (2021a). N6-methyladenosine (m6A)-mediated messenger RNA signatures and the tumor immune microenvironment can predict the prognosis of hepatocellular carcinoma. *Ann. Transl. Med.* 9:59. 10.21037/atm-20-7396 33553352 PMC7859781

[B97] Shen S. Zhang R. Jiang Y. Li Y. Lin L. Liu Z. (2021b). Comprehensive analyses of m6A regulators and interactive coding and non-coding RNAs across 32 cancer types. *Mol. Cancer* 20:67. 10.1186/s12943-021-01362-2 33849552 PMC8045265

[B98] Shi H. Wang X. Lu Z. Zhao B. S. Ma H. Hsu P. J. (2017). YTHDF3 facilitates translation and decay of N(6)-methyladenosine-modified RNA. *Cell Res.* 27 315–328. 10.1038/cr.2017.15 28106072 PMC5339834

[B99] Simone C. N. (2020). First randomized trial supporting the use of proton over photon chemoradiotherapy in esophageal cancer. *J. Clin. Oncol.* 38 2952–2955. 10.1200/JCO.20.01405 32706638

[B100] Su R. Dong L. Li C. Nachtergaele S. Wunderlich M. Qing Y. (2018). R-2HG exhibits anti-tumor activity by targeting FTO/m(6)A/MYC/CEBPA signaling. *Cell* 172 90–105. 10.1016/j.cell.2017.11.031 29249359 PMC5766423

[B101] Su R. Dong L. Li Y. Gao M. Han L. Wunderlich M. (2020). Targeting FTO suppresses cancer stem cell maintenance and immune evasion. *Cancer Cell* 38 79–96.e11. 10.1016/j.ccell.2020.04.017 32531268 PMC7363590

[B102] Tang R. Zhang Y. Liang C. Xu J. Meng Q. Hua J. (2020). The role of m6A-related genes in the prognosis and immune microenvironment of pancreatic adenocarcinoma. *PeerJ* 8:e9602. 10.7717/peerj.9602 33062408 PMC7528816

[B103] Tong J. Wang X. Liu Y. Ren X. Wang A. Chen Z. (2021). Pooled CRISPR screening identifies m(6)A as a positive regulator of macrophage activation. *Sci. Adv.* 7:eabd4742. 10.1126/sciadv.abd4742 33910903 PMC8081357

[B104] Ueda Y. Ooshio I. Fusamae Y. Kitae K. Kawaguchi M. Jingushi K. (2017). AlkB homolog 3-mediated tRNA demethylation promotes protein synthesis in cancer cells. *Sci. Rep.* 7:42271. 10.1038/srep42271 28205560 PMC5304225

[B105] Vaddepally R. K. Kharel P. Pandey R. Garje R. Chandra A. B. (2020). Review of indications of FDA-approved immune checkpoint inhibitors per NCCN guidelines with the level of evidence. *Cancers (Basel)* 12:738. 10.3390/cancers12030738 32245016 PMC7140028

[B106] Van Allen E. M. Miao D. Schilling B. Shukla S. A. Blank C. Zimmer L. (2015). Genomic correlates of response to CTLA-4 blockade in metastatic melanoma. *Science* 350 207–211. 10.1126/science.aad0095 26359337 PMC5054517

[B107] Vom B. J. Vrohlings M. Haller S. Haimovici A. Kulig P. Sledzinska A. (2013). Intratumoral IL-12 combined with CTLA-4 blockade elicits T cell-mediated glioma rejection. *J. Exp. Med.* 210 2803–2811. 10.1084/jem.20130678 24277150 PMC3865478

[B108] Wang G. Dai Y. Li K. Cheng M. Xiong G. Wang X. (2021). Deficiency of mettl3 in bladder cancer stem cells inhibits bladder cancer progression and angiogenesis. *Front. Cell Dev. Biol.* 9:627706. 10.3389/fcell.2021.627706 33681207 PMC7930389

[B109] Wang H. Deng Q. Lv Z. Ling Y. Hou X. Chen Z. (2019). N6-methyladenosine induced miR-143-3p promotes the brain metastasis of lung cancer via regulation of VASH1. *Mol. Cancer* 18:181. 10.1186/s12943-019-1108-x 31823788 PMC6902331

[B110] Wang J. Y. Chen L. J. Qiang P. (2020a). The potential role of N6-methyladenosine (m6A) demethylase fat mass and obesity-associated gene (FTO) in human cancers. *Onco Targets Ther.* 13 12845–12856. 10.2147/OTT.S283417 33364780 PMC7751723

[B111] Wang L. Hui H. Agrawal K. Kang Y. Li N. Tang R. (2020b). M(6) a RNA methyltransferases METTL3/14 regulate immune responses to anti-PD-1 therapy. *EMBO J.* 39:e104514. 10.15252/embj.2020104514 32964498 PMC7560214

[B112] Wang P. Doxtader K. A. Nam Y. (2016). Structural basis for cooperative function of mettl3 and mettl14 methyltransferases. *Mol. Cell* 63 306–317. 10.1016/j.molcel.2016.05.041 27373337 PMC4958592

[B113] Wang Q. Chen C. Ding Q. Zhao Y. Wang Z. Chen J. (2020c). METTL3-mediated m(6)A modification of HDGF mRNA promotes gastric cancer progression and has prognostic significance. *Gut* 69 1193–1205. 10.1136/gutjnl-2019-319639 31582403

[B114] Wang T. Kong S. Tao M. Ju S. (2020d). The potential role of RNA N6-methyladenosine in Cancer progression. *Mol. Cancer* 19:88. 10.1186/s12943-020-01204-7 32398132 PMC7216508

[B115] Wang X. Feng J. Xue Y. Guan Z. Zhang D. Liu Z. (2016). Structural basis of N(6)-adenosine methylation by the METTL3-METTL14 complex. *Nature* 534 575–578. 10.1038/nature18298 27281194

[B116] Wang X. Yang X. Zhang C. Wang Y. Cheng T. Duan L. (2020e). Tumor cell-intrinsic PD-1 receptor is a tumor suppressor and mediates resistance to PD-1 blockade therapy. *Proc. Natl. Acad. Sci. U.S.A.* 117 6640–6650. 10.1073/pnas.1921445117 32161124 PMC7104341

[B117] Wang X. Zhao B. S. Roundtree I. A. Lu Z. Han D. Ma H. (2015). N(6)-methyladenosine modulates messenger RNA translation efficiency. *Cell* 161 1388–1399. 10.1016/j.cell.2015.05.014 26046440 PMC4825696

[B118] Wang Y. Cong R. Liu S. Zhu B. Wang X. Xing Q. (2021). Decreased expression of METTL14 predicts poor prognosis and construction of a prognostic signature for clear cell renal cell carcinoma. *Cancer Cell Int.* 21:46. 10.1186/s12935-020-01738-2 33430867 PMC7802286

[B119] Wanna-Udom S. Terashima M. Lyu H. Ishimura A. Takino T. Sakari M. (2020). The m6A methyltransferase METTL3 contributes to Transforming Growth Factor-beta-induced epithelial-mesenchymal transition of lung cancer cells through the regulation of JUNB. *Biochem. Biophys. Res. Commun.* 524 150–155. 10.1016/j.bbrc.2020.01.042 31982139

[B120] Warda A. S. Kretschmer J. Hackert P. Lenz C. Urlaub H. Hobartner C. (2017). Human METTL16 is a N(6)-methyladenosine (m(6)A) methyltransferase that targets pre-mRNAs and various non-coding RNAs. *EMBO Rep.* 18 2004–2014. 10.15252/embr.201744940 29051200 PMC5666602

[B121] Wei J. Liu F. Lu Z. Fei Q. Ai Y. He P. C. (2018). Differential m(6)A, m(6)Am, and m(1)A demethylation mediated by FTO in the cell nucleus and cytoplasm. *Mol. Cell* 71 973–985. 10.1016/j.molcel.2018.08.011 30197295 PMC6151148

[B122] Wen J. Lv R. Ma H. Shen H. He C. Wang J. (2018). Zc3h13 regulates nuclear RNA m(6)A methylation and mouse embryonic stem cell self-renewal. *Mol. Cell* 69 1028–1038. 10.1016/j.molcel.2018.02.015 29547716 PMC5858226

[B123] World Health Organization (2014). *World Cancer Report 2014*, eds Wild C. P Stewart B. W. (Geneva: World Health Organization).

[B124] Wu X. Sheng H. Wang L. Xia P. Wang Y. Yu L. (2021). A five-m6A regulatory gene signature is a prognostic biomarker in lung adenocarcinoma patients. *Aging* 13 10034–10057. 10.18632/aging.202761 33795529 PMC8064222

[B125] Xiao W. Adhikari S. Dahal U. Chen Y. S. Hao Y. J. Sun B. F. (2016). Nuclear m(6)A reader YTHDC1 regulates mRNA splicing. *Mol. Cell* 61 507–519. 10.1016/j.molcel.2016.01.012 26876937

[B126] Xiao Y. Thakkar K. N. Zhao H. Broughton J. Li Y. Seoane J. A. (2020). The m(6)A RNA demethylase FTO is a HIF-independent synthetic lethal partner with the VHL tumor suppressor. *Proc. Natl. Acad. Sci. U.S.A.* 117 21441–21449. 10.1073/pnas.2000516117 32817424 PMC7474618

[B127] Xu F. Chen J. X. Yang X. B. Hong X. B. Li Z. X. Lin L. (2020a). Analysis of lung adenocarcinoma subtypes based on immune signatures identifies clinical implications for cancer therapy. *Mol. Ther. Oncolytics* 17 241–249. 10.1016/j.omto.2020.03.021 32346613 PMC7183104

[B128] Xu F. Huang X. Li Y. Chen Y. Lin L. (2021a). M(6)A-related lncRNAs are potential biomarkers for predicting prognoses and immune responses in patients with LUAD. *Mol. Ther. Nucleic Acids* 24 780–791. 10.1016/j.omtn.2021.04.003 33996259 PMC8094594

[B129] Xu F. Zhang H. Chen J. Lin L. Chen Y. (2020b). Immune signature of T follicular helper cells predicts clinical prognostic and therapeutic impact in lung squamous cell carcinoma. *Int. Immunopharmacol.* 81:105932. 10.1016/j.intimp.2019.105932 31836430

[B130] Xu F. Zhang Z. Yuan M. Zhao Y. Zhou Y. Pei H. (2021b). M6A regulatory genes play an important role in the prognosis, progression and immune microenvironment of pancreatic adenocarcinoma. *Cancer Invest.* 39 39–54. 10.1080/07357907.2020.1834576 33176521

[B131] Yang S. Wei J. Cui Y. H. Park G. Shah P. Deng Y. (2019). M(6)A mRNA demethylase FTO regulates melanoma tumorigenicity and response to anti-PD-1 blockade. *Nat. Commun.* 10:2782. 10.1038/s41467-019-10669-0 31239444 PMC6592937

[B132] Yang Z. Wang T. Wu D. Min Z. Tan J. Yu B. (2020). RNA N6-methyladenosine reader IGF2BP3 regulates cell cycle and angiogenesis in colon cancer. *J. Exp. Clin. Cancer Res.* 39:203. 10.1186/s13046-020-01714-8 32993738 PMC7523351

[B133] Yao Y. Yang Y. Guo W. Xu L. You M. Zhang Y. C. (2021). METTL3-dependent m(6)A modification programs T follicular helper cell differentiation. *Nat. Commun.* 12:1333. 10.1038/s41467-021-21594-6 33637761 PMC7910450

[B134] Yin H. Zhang X. Yang P. Zhang X. Peng Y. Li D. (2021). RNA m6A methylation orchestrates cancer growth and metastasis via macrophage reprogramming. *Nat. Commun.* 12:1394. 10.1038/s41467-021-21514-8 33654093 PMC7925544

[B135] Yue Y. Liu J. Cui X. Cao J. Luo G. Zhang Z. (2018). VIRMA mediates preferential m(6)A mRNA methylation in 3′UTR and near stop codon and associates with alternative polyadenylation. *Cell Discov.* 4:10. 10.1038/s41421-018-0019-0 29507755 PMC5826926

[B136] Zeng C. Huang W. Li Y. Weng H. (2020). Roles of METTL3 in cancer: mechanisms and therapeutic targeting. *J. Hematol. Oncol.* 13:117. 10.1186/s13045-020-00951-w 32854717 PMC7457244

[B137] Zeng D. Li M. Zhou R. Zhang J. Sun H. Shi M. (2019). Tumor microenvironment characterization in gastric cancer identifies prognostic and immunotherapeutically relevant gene signatures. *Cancer Immunol. Res.* 7 737–750. 10.1158/2326-6066.CIR-18-0436 30842092

[B138] Zeng Y. Xiang Y. Sheng R. Tomas H. Rodrigues J. Gu Z. (2021). Polysaccharide-based nanomedicines for cancer immunotherapy: a review. *Bioact. Mater.* 6 3358–3382. 10.1016/j.bioactmat.2021.03.008 33817416 PMC8005658

[B139] Zhang B. Wu Q. Li B. Wang D. Wang L. Zhou Y. L. (2020). M(6)A regulator-mediated methylation modification patterns and tumor microenvironment infiltration characterization in gastric cancer. *Mol. Cancer* 19:53. 10.1186/s12943-020-01170-0 32164750 PMC7066851

[B140] Zhang C. Huang S. Zhuang H. Ruan S. Zhou Z. Huang K. (2020). YTHDF2 promotes the liver cancer stem cell phenotype and cancer metastasis by regulating OCT4 expression via m6A RNA methylation. *Oncogene* 39 4507–4518. 10.1038/s41388-020-1303-7 32366907

[B141] Zhang C. Samanta D. Lu H. Bullen J. W. Zhang H. Chen I. (2016). Hypoxia induces the breast cancer stem cell phenotype by HIF-dependent and ALKBH5-mediated m(6)A-demethylation of NANOG mRNA. *Proc. Natl. Acad. Sci. U.S.A.* 113 E2047–E2056. 10.1073/pnas.1602883113 27001847 PMC4833258

[B142] Zhang C. Zhang M. Ge S. Huang W. Lin X. Gao J. (2019). Reduced m6A modification predicts malignant phenotypes and augmented Wnt/PI3K-Akt signaling in gastric cancer. *Cancer Med.* 8 4766–4781. 10.1002/cam4.2360 31243897 PMC6712480

[B143] Zhao H. Xu Y. Xie Y. Zhang L. Gao M. Li S. (2021). M6A regulators is differently expressed and correlated with immune response of esophageal cancer. *Front. Cell Dev. Biol.* 9:650023. 10.3389/fcell.2021.650023 33748145 PMC7970005

[B144] Zhao Q. Zhao Y. Hu W. Zhang Y. Wu X. Lu J. (2020). M(6)A RNA modification modulates PI3K/Akt/mTOR signal pathway in gastrointestinal cancer. *Theranostics* 10 9528–9543. 10.7150/thno.42971 32863943 PMC7449908

[B145] Zheng G. Dahl J. A. Niu Y. Fedorcsak P. Huang C. M. Li C. J. (2013). ALKBH5 is a mammalian RNA demethylase that impacts RNA metabolism and mouse fertility. *Mol. Cell* 49 18–29. 10.1016/j.molcel.2012.10.015 23177736 PMC3646334

[B146] Zheng W. Dong X. Zhao Y. Wang S. Jiang H. Zhang M. (2019). Multiple functions and mechanisms underlying the role of METTL3 in human cancers. *Front. Oncol.* 9:1403. 10.3389/fonc.2019.01403 31921660 PMC6920212

[B147] Zhong J. Liu Z. Cai C. Duan X. Deng T. Zeng G. (2021). M(6)A modification patterns and tumor immune landscape in clear cell renal carcinoma. *J. Immunother. Cancer* 9:e001646. 10.1136/jitc-2020-001646 33574053 PMC7880120

[B148] Zhou K. I. Shi H. Lyu R. Wylder A. C. Matuszek Z. Pan J. N. (2019). Regulation of co-transcriptional Pre-mRNA splicing by m(6)A through the low-complexity protein hnRNPG. *Mol. Cell* 76 70–81. 10.1016/j.molcel.2019.07.005 31445886 PMC6778029

[B149] Zhou Z. Zhang J. Xu C. Yang J. Zhang Y. Liu M. (2021). An integrated model of N6-methyladenosine regulators to predict tumor aggressiveness and immune evasion in pancreatic cancer. *EBioMedicine* 65:103271. 10.1016/j.ebiom.2021.103271 33714027 PMC7966986

[B150] Zhuang C. Zhuang C. Luo X. Huang X. Yao L. Li J. (2019). N6-methyladenosine demethylase FTO suppresses clear cell renal cell carcinoma through a novel FTO-PGC-1alpha signalling axis. *J. Cell. Mol. Med.* 23 2163–2173. 10.1111/jcmm.14128 30648791 PMC6378205

